# An overview of biochar production techniques and application in iron and steel industries

**DOI:** 10.1186/s40643-024-00779-z

**Published:** 2024-07-03

**Authors:** Segun E. Ibitoye, Chanchal Loha, Rasheedat M. Mahamood, Tien-Chien Jen, Meraj Alam, Ishita Sarkar, Partha Das, Esther T. Akinlabi

**Affiliations:** 1 Department of Mechanical Engineering, Faculty of Engineering and Technology, University of Ilorin, P. M. B. 1515, Ilorin, Nigeria; 2 School of Engineering, Woxsen University, Kamkole Village, Sadasivpet, Sangareddy District, Hyderabad, Telangana 502345 India; 3 Energy Research and Technology Group, CSIR-Central Mechanical Engineering Research Institute, Durgapur, West Bengal 713209 India; 4 Department of Mechanical Engineering Science, Faculty of Engineering and the Built Environment, University of Johannesburg, P. O. Box 524, Auckland Park, 2006 South Africa; 5 Department of Mechanical and Construction Engineering, Faculty of Engineering and Environment, Northumbria University, Newcastle, NE1 8ST UK

**Keywords:** Biochar, Biomass conversion, Carbon sequestration, Environmental responsibility, Iron and steel industries

## Abstract

Integrating innovation and environmental responsibility has become important in pursuing sustainable industrial practices in the contemporary world. These twin imperatives have stimulated research into developing methods that optimize industrial processes, enhancing efficiency and effectiveness while mitigating undesirable ecological impacts. This objective is exemplified by the emergence of biochar derived from the thermo-chemical transformation of biomass. This review examines biochar production methods and their potential applications across various aspects of the iron and steel industries (ISI). The technical, economic, and sustainable implications of integrating biochar into the ISI were explored. Slow pyrolysis and hydrothermal carbonization are the most efficient methods for higher biochar yield (25–90%). Biochar has several advantages- higher heating value (30–32 MJ/kg), more porosity (58.22%), and significantly larger surface area (113 m^2^/g) compared to coal and coke. However, the presence of biochar often reduces fluidity in a coal-biochar mixture. The findings highlighted that biochar production and implementation in ISI often come with higher costs, primarily due to the higher expense of substitute fuels compared to traditional fossil fuels. The economic viability and societal desirability of biochar are highly uncertain and vary significantly based on factors such as location, feedstock type, production scale, and biochar pricing, among others. Furthermore, biomass and biochar supply chain is another important factor which determines its large scale implementation. Despite these challenges, there are opportunities to reduce emissions from BF-BOF operations by utilizing biochar technologies. Overall, the present study explored integrating diverse biochar production methods into the ISI aiming to contribute to the ongoing research on sustainable manufacturing practices, underscoring their significance in shaping a more environmentally conscious future.

**Supplementary Information:**

The online version contains supplementary material available at 10.1186/s40643-024-00779-z.

## Introduction

**Fig. 1:**
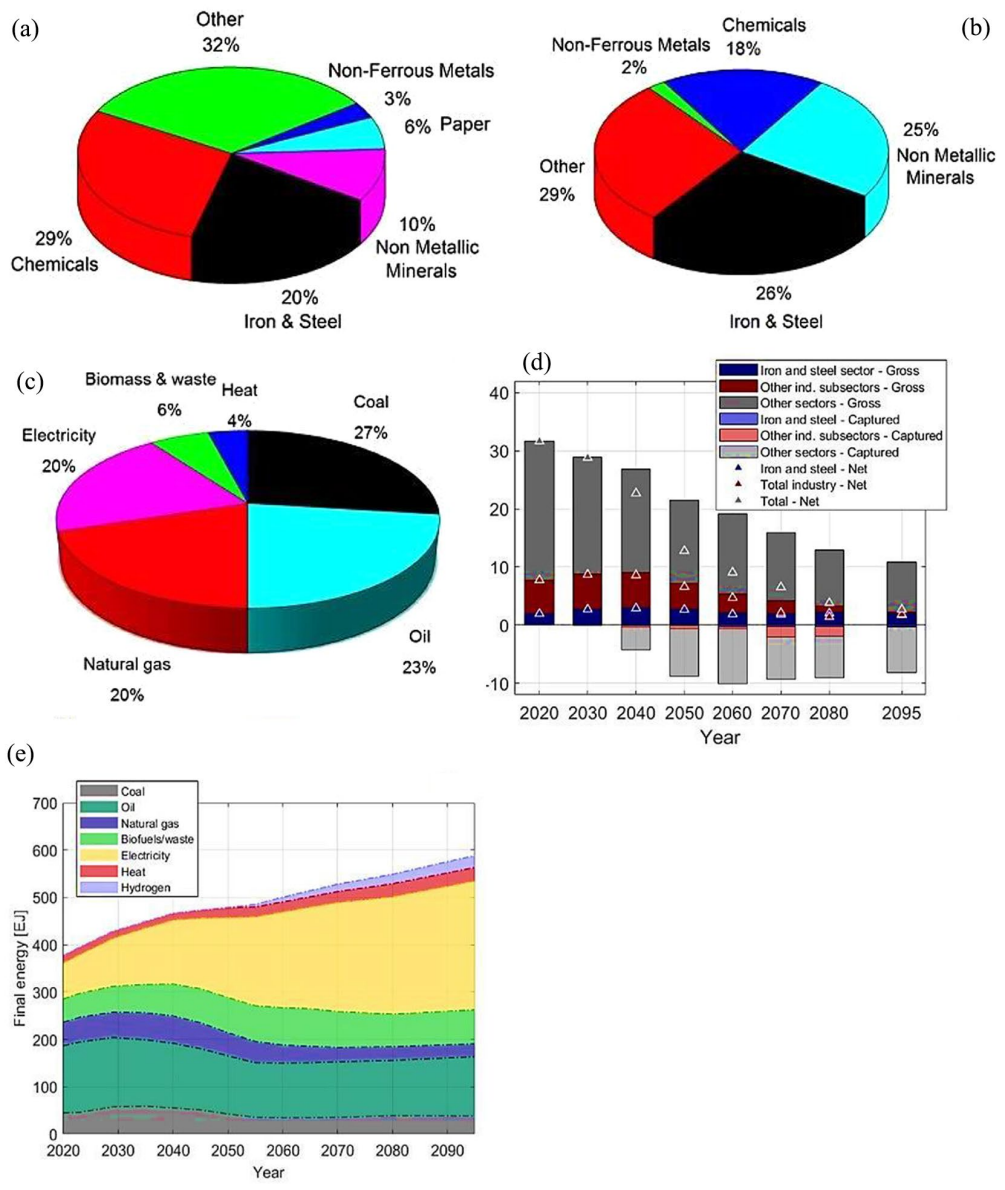
Energy utilization and CO_2_ emission in the industrial sector- (**a**) energy use, (**b**) CO_2_ emission, (**c**) energy consumption by fuel type, (**d**) Energy utilization for all technology, and (**e**) CO_2_ emission for all technology. Reprinted from Moglianesi et al. (2023) and Mousa et al. (2016) (Copyright © 2024, with permission from Elsevier)

The iron and steel industries (ISI) play a significant role in global economic growth and are known for their high energy consumption. As presented in Fig. 1, around 26% of the energy used by industries worldwide is consumed by the ISI, with coal and coke playing a key role (Mousa et al. 2016; Safarian 2023b). Fossil fuels are primarily used to generate heat and as reducing agents in the steel-making process, which results in significant worldwide CO_2_ emissions (Ibitoye 2018; Osman et al. 2022; Sundberg et al. 2020). Studies have shown that using fossil-based carbon during steel-making is responsible for about 60–70% of the CO_2_ emitted in steel production via electric arc furnaces (EAF) and reheating furnaces (Robinson et al. 2021). Also, the dwindling fossil fuel supply is unfavorable to the ISI. These situations motivate the search for reliable, sustainable, and environmentally friendly fuels to replace coal and coke. Biomass sources seem to be one of the promising solutions (Adekunle et al. 2019; Hu et al. 2019; Suopajärvi et al. 2018). The carbon contents of lignocellulose biomasses are high and could be converted into usable energy. Therefore, biomass and biomass residues are thermo-chemically transformed into bio-oil, syngas, and biochar to improve their fuel qualities for various applications in the ISI (Zakaria et al. 2023). Biochar has recently been considered a potential replacement for coal/coke since it can be easily adapted and has qualities equivalent to coal and coke in the metallurgical process. However, the factors limiting biochar application in ISI include cost-effectiveness in large-scale biochar production, challenges in ensuring consistent biochar quality, and integration of biochar into complex processes of iron and steel production (Mousa et al. 2016a).

Biochar is a porous black solid derived from the thermochemical transformation of biomass materials. It is characterized by a high surface area, possessing exceptional physical and chemical attributes that facilitate long-term environmental carbon storage (Reddy et al. 2019; Safarian 2023a). The distinctive characteristics of biochar, encompassing a notable adsorption capacity and ion exchange capability, extend its utility to various applications (Amer et al. 2022; Cho et al. 2023; Majumder et al. 2023). Employing biochar for iron and steel production holds considerable attraction, especially for nations endowed with ample and sustainable biomass resources (Ye et al. 2019; Zaini et al. 2023). This is underpinned by its renewable nature, widespread availability, and versatile applicability (Hamidzadeh et al. 2023; Tan 2023). Pursuing sustainable industrial practices incorporating innovation and environmental responsibility has recently become popular (Chang et al. 2023; Simmou et al. 2023). These two imperatives have sparked research into cutting-edge approaches that improve the efficiency and efficacy of industrial processes while reducing their adverse environmental effects (Le 2022).

Biochar comprehensively addresses climate change, ranging from its function in soil enrichment to its potential integration inside the steel industry, a classic sector distinguished by its significant carbon footprint and complex manufacturing methods (Abhi et al. 2023; Azzi et al. 2022). In light of this context, the steel sector is a top prospect for creative biochar integration. The steel industry seeks solutions to coordinate its operations with sustainable practices because it contributes significantly to global carbon emissions. The ability of biochar to sequester carbon, as shown by its use in land recovery operations, presents an attractive opportunity for the sector.

The potential use of biochar as a reducing agent in steel-making processes is another interesting direction to pursue (Gan et al. 2023; Zhang et al. 2022). The renewable nature of biochar and its ability to operate as a reducing agent provides a solution to lessen dependency on fossil-based reducing agents, promoting a more environmentally friendly steel production cycle. Biochar production from biomass feedstock is another method of managing biomass waste and the problems associated with its disposal. This aligns with the circular economy of converting wastes into usable products (Adeniyi et al. 2023; Chaturvedi et al. 2023; Ismail et al. 2023).

This study examines diverse biochar production methods, observing the possible interactions between the technical and economic practicality of integrating biochar into ISI. Moreover, the technological advancements and real-world implications of integrating biochar in the ISI were examined. The study seeks to give readers an in-depth understanding of the vigorous interaction between biochar production techniques and their potential utilization in the steel industry. Finally, the paper pays attention to the sustainable production practices within the ISI, which is essential for achieving more eco-friendly results.

This paper is organized into 11 sections. The review methodology is presented in Sect. 2. Section 3 discussed the significance of biochar in steel industrial sustainability. The various biochar production techniques are presented in Sect. 4. Section 5 presents technical viability and adaption issues, respectively. The cost implication, scalability, and long-term sustainability of biochar production and use in ISI in discussed in Sect. 6. Section 7 discusses the regulatory and environmental implications. Section 8 presents recent studies on the use of biochar in the ISI. Section 9 presents the biochar integration approach for the ISI. Sections 10 and 11 of the article presented the direction for future studies and conclusions, respectively.

## Review methodology

A thorough search strategy was formulated to discover studies that were pertinent to the review process. This strategy primarily involved searching the ScienceDirect and SpringerLink databases for articles related to biochar production techniques and their applications in the ISI. Additionally, some articles obtained through Google Scholar searches, which were directly relevant but not available in ScienceDirect and SpringerLink, were also considered. The search strategy encompassed a wide range of keywords and combinations to ensure thorough coverage of the subject. These search terms include thermochemical conversion of biomass, biomass torrefaction, biomass pyrolysis, biomass carbonization, biomass gasification, hydrothermal carbonization of biomass, plasma pyrolysis of biomass, slow pyrolysis of biomass, microwave pyrolysis of biomass, biochar production methods, biochar production techniques, biochar application in iron and steel, iron and steel production processes, biochar use in iron and steel industries, coke-making, sintering process, direct reduced iron, biochar co-firing, biochar and coal blend in iron and steel production, biochar use in blast furnace, biochar use electric arc furnace, biochar use as a reducing agents, biochar use for soil remediation, biochar use for climate change reduction, biochar use for CO_2_ reduction, CO_2_ emissions in the industrial sector, significance of biochar in steel industrial sustainability, comparison of biochar characteristics with coal and coke, biochar porosity, biochar surface area, Brunauer-Emmett-Teller surface area of biochar, morphological properties of biochar, microstructural properties of biochar, biochar injection in blast furnaces, biochar utilization in iron ore sintering, biochar use as a foaming agent, biochar yield, higher heating value of biochar, calorific value of biochar efficiency metrics in biochar production, biochar foaming reactivity characteristics, biochar as an alternative to coke breeze, biochar reduction properties, potential for greenhouse gas reduction, coal substitution/co-combustion with biochar, and biochar.

### Inclusion and exclusion criteria

The criteria for inclusion and exclusion of studies were developed based on the subject of the review, drawing from guidelines outlined by Vlachokostas et al. (2021) and Balali et al. (2023). The inclusion criteria include studies published in peer-reviewed journals, studies conducted on the application of biochar in the ISI, studies identifying different biochar production methods and applications, papers investigating the impact of production methods on the properties of biochar, and studies related to iron and steel production. Conversely, the exclusion criteria encompassed investigations that did not meet the inclusion criteria, papers not written in English, manuscripts not available in full text, and studies not carried out in the last seven years. However, some articles published later than 2018, which were directly relevant to the subject, were still included in the review. The specific subject of the review was instrumental in developing data extraction and analysis methods. Data extraction involved identifying relevant information from the included studies, such as the properties of biochar, the production methods employed, biochar application in iron and steel making, environmental implication of biochar, and other beneficial application of biochar.

### Data analysis and evaluation

The evaluation and analysis method entailed synthesizing data from the included research and detecting patterns and trends in the results and discussions. According to Araújo et al. (2020), the quality and validity of the review were guaranteed by adhering to established principles like the Preferred Reporting Items for Systematic Reviews and Meta-Analyses (PRISMA) to ensure transparency and rigor in the review process.

## Significance of biochar in steel industrial sustainability

The creation of biochar offers an exceptional chance for the steel industry to apply circular economy principles. Biochar can be created as a usable product using biomass waste, such as wood scraps and agricultural wastes, for steel-making purposes (Dermawan et al. 2022; Selvarajoo et al. 2022; Tan 2023). This reduces the price of waste/residue disposal while simultaneously optimizing resource use and promoting economic models that are driven by sustainability.

Due to resource depletion and price swings, the steel sector may be vulnerable due to its reliance on fossil-based carbon sources. Diversifying the carbon input stream by including biochar as an alternate carbon source increases resilience against supply disruptions and erratic market dynamics and reduces the carbon emissions of the industry.

The physico-chemical properties of biochar can affect the effectiveness of the steel-making process. Biochars exhibit higher volatile matter (VM) content when compared to coal and coke; however, biochar possesses lower ash content (AC) and fewer impurities, advantageous qualities for iron-making purposes (Azzi et al. 2022; Safarian 2023b). Its capability to absorb impurities can result in cleaner reactions, practically minimizing the presence of some impurities in the molten metal (Safarian 2023b). This cleansing results in higher-quality steel and additional effective processing. Also, enhanced reaction kinetics and process conditions due to biochar utilization minimize the energy use in the ISI. This is because obtaining the preferred results at a lower energy input is possible when reactions continue more efficiently and rapidly. This results in lower energy use per unit of steel, minimizing the total cost of production (Ye et al. 2019).

Biochar exhibits remarkable similarities to coal and coke across various properties, making it a compelling substitute for applications in iron and steelmaking processes. These similarities are detailed in Table 1. Typically, biochar maintains a moisture content (MC) ranging from 1 to 5%, falling within the range of coke (1–10%) and notably lower than coal (10–15%) (Kemppainen et al. 2017; Khanna et al. 2019). This low MC ensures efficient combustion and minimizes energy loss during processing. With a VM content of approximately 10–12%, biochar closely compares to coke (1–2%) and falls below the VM levels found in coal (15–30%) (Kemppainen et al. 2017; Khanna et al. 2019). The moderate VM content enhances the combustibility and energy yield of biochar. Furthermore, biochar contains a high fixed carbon (FC) content, ranging from 85 to 87%, similar to coke (85–88%) and significantly surpassing coal (50–55%) (Kemppainen et al. 2017; Khanna et al. 2019). The high FC ensures a robust energy source and facilitates efficient reduction reactions in iron and steelmaking processes. As depicted in Table 1, biochar exhibits low AC, around 3%, comparable to coal, with an AC of 0.4% and significantly lower than coke, with an AC of 13% (Kemppainen et al. 2017; Khanna et al. 2019). This minimal AC minimizes impurities and residues in the production process, contributing to cleaner and more efficient operations.

**Table 1:** Comparison of biochar characteristics with coal and coke (Gan et al. 2017; Kemppainen et al. 2017; Khanna et al. 2019; safarian 2023b; Zhao and Wei 2022)

Fuel property	Biochar	Coal	Coke
MC (%)	1–5	10–15	1–10
VM (%)	10–12	15–30	1–2
FC (%)	85–87	50–55	85–88
AC (%)	1–2	10	8–12
Mineral matter (%)	1–2	10	8–12
**Bulk density (kg/m** ^**3**^ **)**	180–240	800–850	400–500
HHV (MJ/kg)	30–32	23–28	30
Porosity (%)	58	10	2
**Surface Area (m** ^**2**^ **/g)**	113	4	4
Milling requirement	Standard	Standard	N/A
Transportation Costs	Medium	Medium	Low

With a relatively low mineral matter content ranging from 1 to 1.4%, biochar closely aligns with coke (8–12%) and exhibits substantially less mineral matter than coal (10%). This reduced mineral content enhances the purity and quality of biochar for industrial applications. In addition, biochar possesses a lower bulk density compared to coal and coke, ranging from 180 to 240 kg/m^3^, while coal and coke have bulk densities of 800–850 kg/m^3^ and 400–500 kg/m^3^, respectively (Kemppainen et al. 2017; Khanna et al. 2019). The lower bulk density of biochar may impact transportation and handling considerations but offers advantages in terms of porosity and reactivity. Biochar displayed a higher heating value ranging from 30 to 32 MJ/kg, aligning closely with coke (30 MJ/kg) and surpassing coal (23 MJ/kg). This elevated heating value underscores the energy-rich nature of biochar, making it a potent fuel source for iron and steelmaking processes.

Biochar exhibits high porosity, approximately 58%, which significantly exceeds the porosity levels found in coal (10%) and coke (2.47%) (Gan et al. 2017; Zhao and Wei 2022). This porosity provides ample surface area for contact between the reducing agent and iron oxides, facilitating the reaction kinetics. Additionally, the surface area of biochar allows for the adsorption of gases, such as CO_2_ and CO, further enhancing its reactivity in the reduction process. Similarly, biochar demonstrates a large surface area, approximately 113 m^2^/g, significantly surpassing the surface areas of coal and coke with surface areas of 4 m^2^/g.

Biochar shares standard milling requirements with coal, ensuring ease of processing and compatibility with existing infrastructure. Both biochar and coal incur medium transportation costs, reflecting similar considerations in logistics and distribution. In contrast, coke generally requires lower transportation costs due to its higher bulk density.

**Fig. 2:**
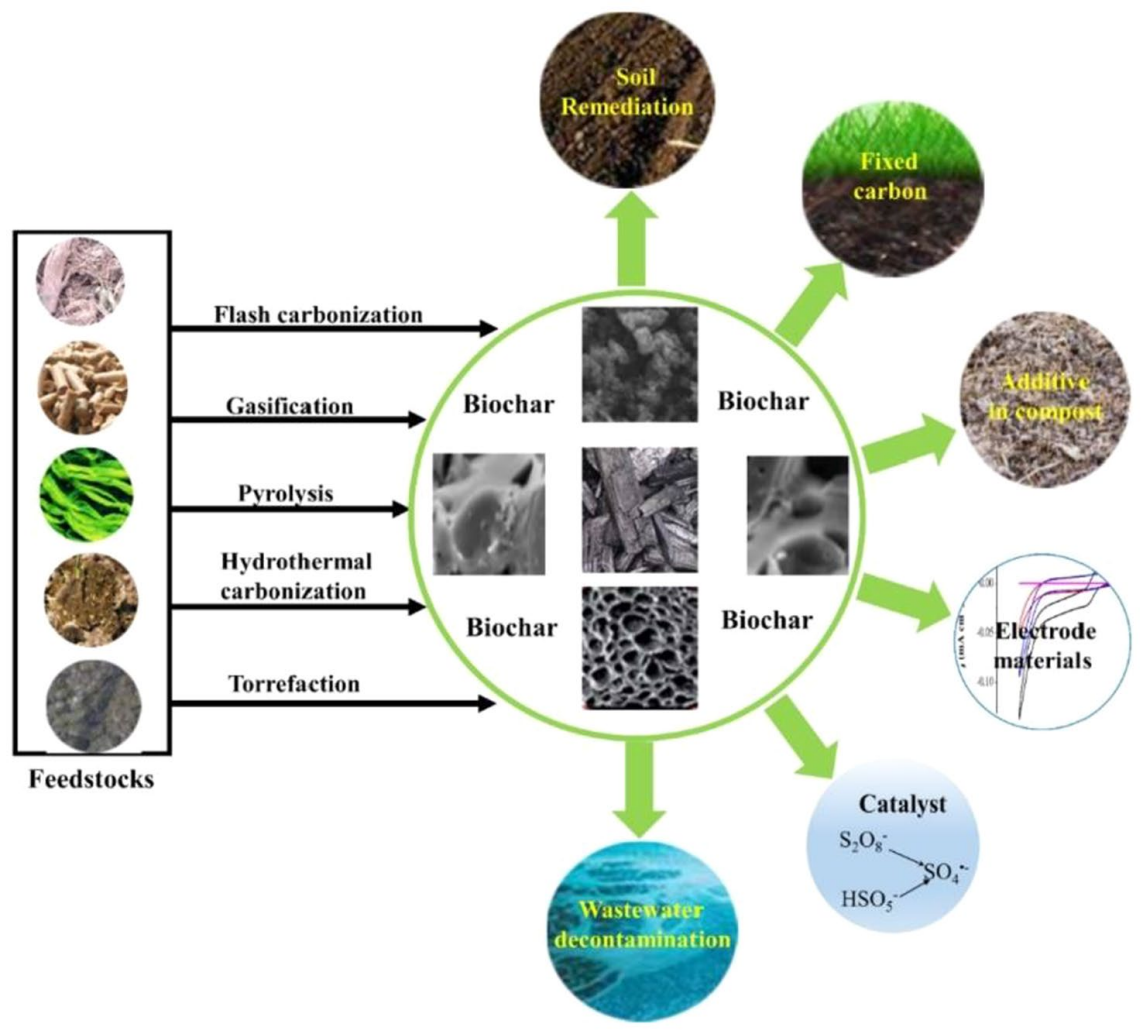
Different applications of biochar. Reprinted from Wang and Wang (2019) (Copyright © 2024, with permission from Elsevier)

Biochar has numerous applications within the ISI that could offer innovative solutions to persistent environmental challenges and enhance productivity and sustainable practices (Fig. 2). The possible applications of biochar in the ISI are enumerated as follows:

i. Reducing agent: Biochar can be used as a reducing agent as a substitute for conventional carbon-based sources like coal (Dermawan et al. 2022; Selvarajoo et al. 2022; Tan 2023). It can serve as a reducing agent in direct reduction or blast furnace iron-making to convert iron oxides into metallic iron. This application can help lower the CO_2_ emissions linked to ISI. The reduction characteristics of biochar is further discussed in Sect. 4.4.
ii. Heat and energy generation: Due to its high calorific values and carbon content, biochar can generation heat or be used in energy recovery processes in the ISI. The calorific value of biochar can be utilized for different energy needs by burning it or co-firing with other fuels (Ibitoye et al. 2023a, b).
iii. Carbon sequestration: Biochar is crucial in mitigating climate change by sequestering carbon (Danesh et al. 2023). Integrating biochar in iron and steel industrial processes, such as reforestation efforts, can help sequester CO_2_ from the atmosphere and reduce emissions (Farghali et al. 2022; Sivaranjanee et al. 2023). The ISI can help in carbon capture efforts by adding biochar-enriched soils into their operation, compensating its CO_2_ emissions, and supplementing the general climate change alleviation plans.
iv. Waste management: The production of biochar from industrial, domestic, agricultural, livestock, forest, and municipal solid wastes, among others, serves as a waste management technique (Abhi et al. 2023; Xu et al. 2021). Several studies on generating biochar form wastes/residues have been reported in the literature. The biochar production from food waste, rice husk, and grape tree branch waste has been investigated via slow pyrolysis in the CO_2_ and N_2_ atmosphere (Premchand et al. 2023b). Results showed that CO_2_ increased biochar yield and influenced its properties significantly, indicating CO_2_’s potential for tailored biochar production from various waste sources.
  In Gondar, laboratory studies investigated the impact of airflow rate, heating rate, temperature, and residence time on biochar yields from cud and waste paper during slow pyrolysis. The research revealed that temperature and airflow rate are the primary factors influencing the quantity of biochar produced. At 167 °C, cud and waste paper produced different biochar amounts, yet higher airflow rates and temperatures led to decreased biochar yields (Nega et al. 2023).
  Improving soil quality: ISI is known to possess large areas of land. Therefore, biochar can enhance soil quality and fertility in land recovery activities (Osman et al. 2022; Zhang et al. 2012).
  Contaminated soils containing heavy metals threaten global food safety by hampering plant growth and reducing crop yields. With an increasing population and food demand, finding efficient, cost-effective, and environmentally friendly soil remediation methods is imperative. Among various options, the utilization of biochar stands out due to its effectiveness, affordability, and minimal ecological impact (Mehmood et al. 2023). The biochar materials work by reducing the availability of metals in the soil, thereby enhancing crop outputs. Research has revealed that biochar enhances soil fertility, improves soil structure, and boosts agricultural yields (Danesh et al. 2023). A field study conducted in Henan, central China, investigated the impact of biochar generation on corn yield in soil with low organic carbon (Zhang et al. 2012). Biochar was applied at 0, 20, and 40 tons per hectare, with or without nitrogen fertilization. Results indicated that biochar significantly increased maize yield, particularly at the higher application rates.
  The preparation, analysis techniques, and biochar applications have been studied, emphasizing its significant role in agriculture and related sectors (Hamidzadeh et al. 2023; Pourhashem et al. 2019). Specifically, the study underscores biochar’s potential as a sustainable fertilizer. Reports also showed that biochar enriches the soil, shields microorganisms from unfavorable conditions, and influences soil pH and microbial community activity, thereby sustainably enhancing agricultural productivity (Osman et al. 2022; Tan 2023).
  A detailed review has examined the potential of converting biomass waste into biochar with improved nutritional qualities for agricultural applications (Tan 2023). The report showed that biochar enhances the physico-chemical characteristics of soil, aiding in the retention of minerals and water, and thereby boosting soil fertility.
  The characteristics and agricultural uses of biochar depend heavily on how it is made and the feedstock used. Process temperature and feedstock composition influence its effectiveness in enhancing soil and promoting plant growth (Gabhane et al. 2020).
v. Enhancing flux and slag qualities: The morphological properties (porous nature and reactivity) of biochar can influence the flux and slag qualities in the steel-making process. Biochar utilization can result in lessened refractory degradation, improved desulfurization, and slag production (Mehmood et al. 2023; Reddy et al. 2019).
vi. Water quality improvement: Biochar is crucial in water and wastewater treatments, effectively eliminating contaminants such as organic, heavy metals, pesticides, dyes, and inorganic materials (Li et al. 2023; Saha and Sengupta 2021). Shikuku et al. (2018) utilized biochar as an eco-friendly and economical adsorbent for purifying wastewater systems by removing organic pollutants and controlling microbial growth.
  A comprehensive review compares the biochar characteristics of various biomass and plastic wastes, highlighting that feedstock composition and reactor setup influence the resulting biochar properties (Adeniyi et al. 2023). The report showed that the resulting biochar from biomass and plastic finds application in water treatment. The review underscores the need for technological advancements, economic benefits, and increased government involvement and public awareness to promote the utilization of biochar for water and waste treatment purposes.

**Fig. 3:**
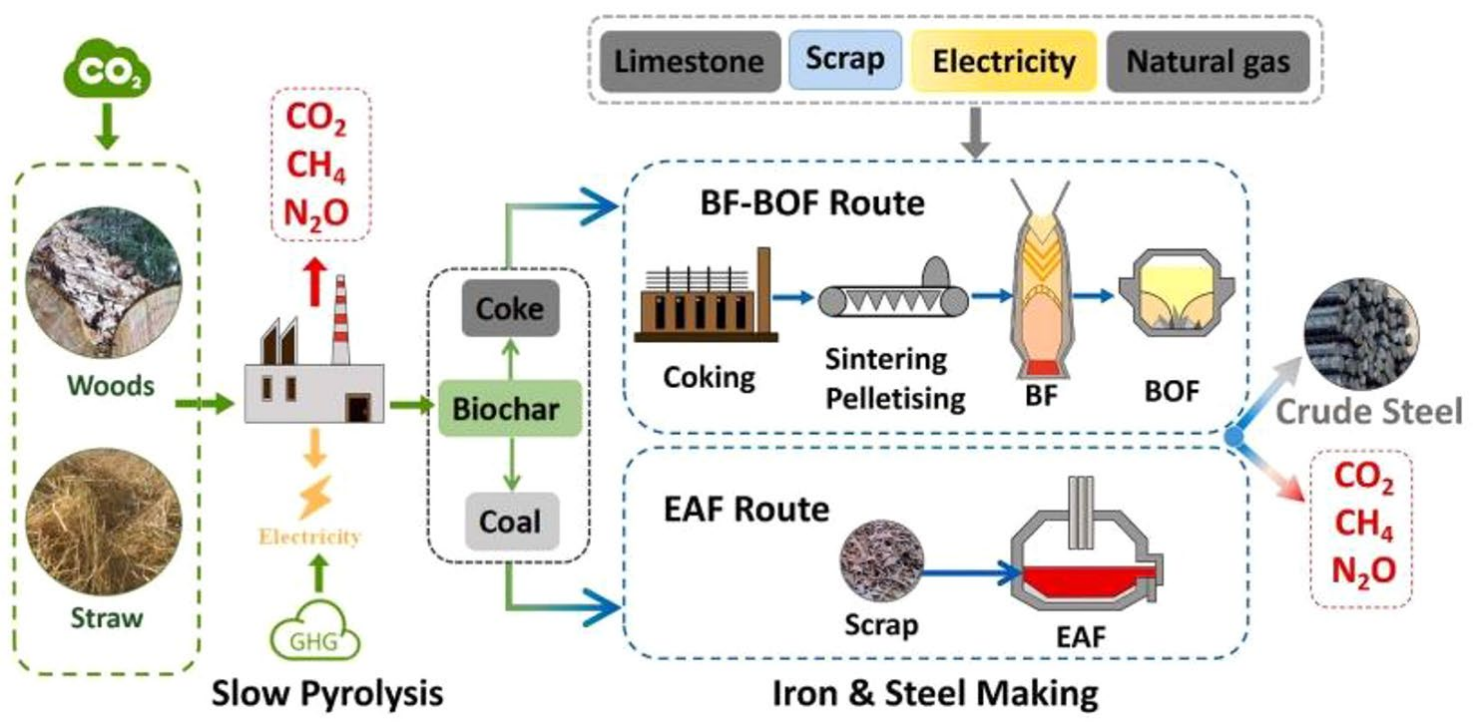
Biochar use in an integrated ISI. Reprinted from Meng et al. (2024) (Copyright © 2024, with permission from Elsevier)

A typical utilization of biochar in ISI is displayed in Fig. 3. Integrating biochar into steel manufacturing methods demonstrates a commitment to sustainability and environmental stewardship. This is also known as corporate social responsibility. These initiatives can boost the industry’s reputation, attract environmentally conscious investors, and help industries achieve social responsibility objectives.

### Utilization of biochar in coke making

Coke plays several crucial roles in blast furnace (BF) iron-making (Cirilli et al. 2018). Its primary role within a blast furnace involves providing fuel to generate heat energy for the chemical processes and the liquefaction of slag and metal. Furthermore, it serves as a reducing agent and carburizer for the molten metal in the hearth. Coke provides a support medium for the iron-containing load, forming a porous structure for liquid slag and hot metal (Zhang et al. 2022).

Up to 15% biochar can be added to coal blends to produce coke of sufficient quality. Fluidity, crucial for coke quality, reflects a coal’s ability to form a plastic phase, ranging from 1 to 5000 ddpm (Khanna et al. 2019). Biochars don’t transition to the plastic phase during cooking; thus, their addition typically decreases maximum fluidity in coal-biochar blends.

Biochars undergo thermal decomposition at temperatures similar to coal, happening during the transition from semi-coke to coke. While remaining comparatively unreactive during melting processes, biochar plays a crucial role in binding various plastic phases. Its addition to coal blends affects coke matrix formation and stability.

The coke strength is assessed via shatter and drum tests, with post-reaction strength measured using the Coke Strength after Reaction (CSR) index, linked to the Coke Reactivity Index (CRI) (Ajimotokan et al. 2019a, b; Alvarez et al. 2007). A high CSR value enhances coke durability, gas and liquid permeability, and productivity, reducing exact coke ingestion in BFs. BFs typically need cokes with CRI and CSR within value ranges of 20–30 and 58–65, respectively (Alvarez et al. 2007). Incorporating biochars into coal blends impacts coke reactivity. Reactivity generally increases with higher biochar levels. However, a study revealed that the addition of pine sawdust char up to 5% can keep CRI values below 30, while chestnut and pine sawdust chars (1–5 wt%) result in increased CRI and decreased CSR (Montiano et al. 2014). Industrially viable CRI and CSR ranges necessitate biochar additions below 2 wt%.

### Utilization of biochar in iron ore sintering

Sintering iron ore in BF ironmaking commonly involves blending coke breeze (up to 3–5 wt%) and fluxes like dolomite, limestone, and silica to form pellets. Biochar fuels can partially replace coke breeze, but their higher reactivity due to porosity and surface area may impact sintering and quality. Ooi et al. (2008) studied substituting coke with sunflower husk chars in sintering. Various biochars were tested as sintering fuels, with wood char showing satisfactory results. Substituting coke breeze with biochar increased sintering speed but reduced yields and productivity at higher substitution rates (El-Hussiny et al. 2015; Mousa et al. 2015). Biochars also decreased sinter bulk density, facilitated quicker combustion temperatures, and led to thinner combustion and sintering zones in blast furnaces. However, they resulted in lower sinter strength. Biochar’s share in iron ore sinters is restricted to < 25 wt% (El-Hussiny et al. 2015; Mousa et al. 2015).

### Injection of biochar in the blast furnace

Studies have been conducted to investigate biochar injection in BFs, focusing on material handling and grinding properties (Gil et al. 2015; Pohlmann et al. 2016). Gil et al. (2015) investigated the grindability of chest wood, poplar, and pine at 240–300 ^o^C, and an improved grindability of biochars with higher process temperature and longer dwelling time was reported. Combining coal with torrefied chestnut chips as an injectant showed minimal interaction. Pine biochar behaved similarly to coal, allowing biochar proportions in blends without adverse effects. Pohlmann et al. (2016) examine the flammability of eucalyptus compressed at varying temperatures compared to coals with comparable VM contents frequently utilized for PCI in BFs. The results showed an increase in burnout of biochars compared to coals. Industrial trials and models suggest that injecting pulverized biochar particles into BFs significantly reduces CO_2_ emissions. However, replacing coals with biochars may increase operating costs (Gil et al. 2015; Pohlmann et al. 2016).

### Utilization of biochar as a foaming agent

Utilizing biochars in EAF is more straightforward than BF processes due to EAF’s batch nature and quick turnovers. Biochar-based direct reduced iron (DRI) can partly substitute feedstock during charging, and biochars can serve as slag foaming agents, alone or in blends with coke. It has been emphasized that the FC, AC, and VM, calorific value, and reactivity role are crucial parameters for biochar application steel-making (Cirilli et al. 2018; Salimbeni et al. 2023). High-reactivity biochars enhance slag foaming but may require briquetting for adequate carbon transfer into slag (Bianco et al. 2013). Plant trials with biochar fines didn’t negatively impact steel quality or slag foaming, but molten iron carburization was suboptimal due to rapid biochar combustion (Bianco et al. 2013). Industrial-scale trials in EAFs showed no substantial discrepancies in slag and metal quality compared to coal (Demus et al. 2012). However, handling issues, scattering of low-density powders, and concentrated flame emissions were observed during biochar trials, with limited slag foaming due to biochar penetration.

## Biochar production techniques

**Fig. 4:**
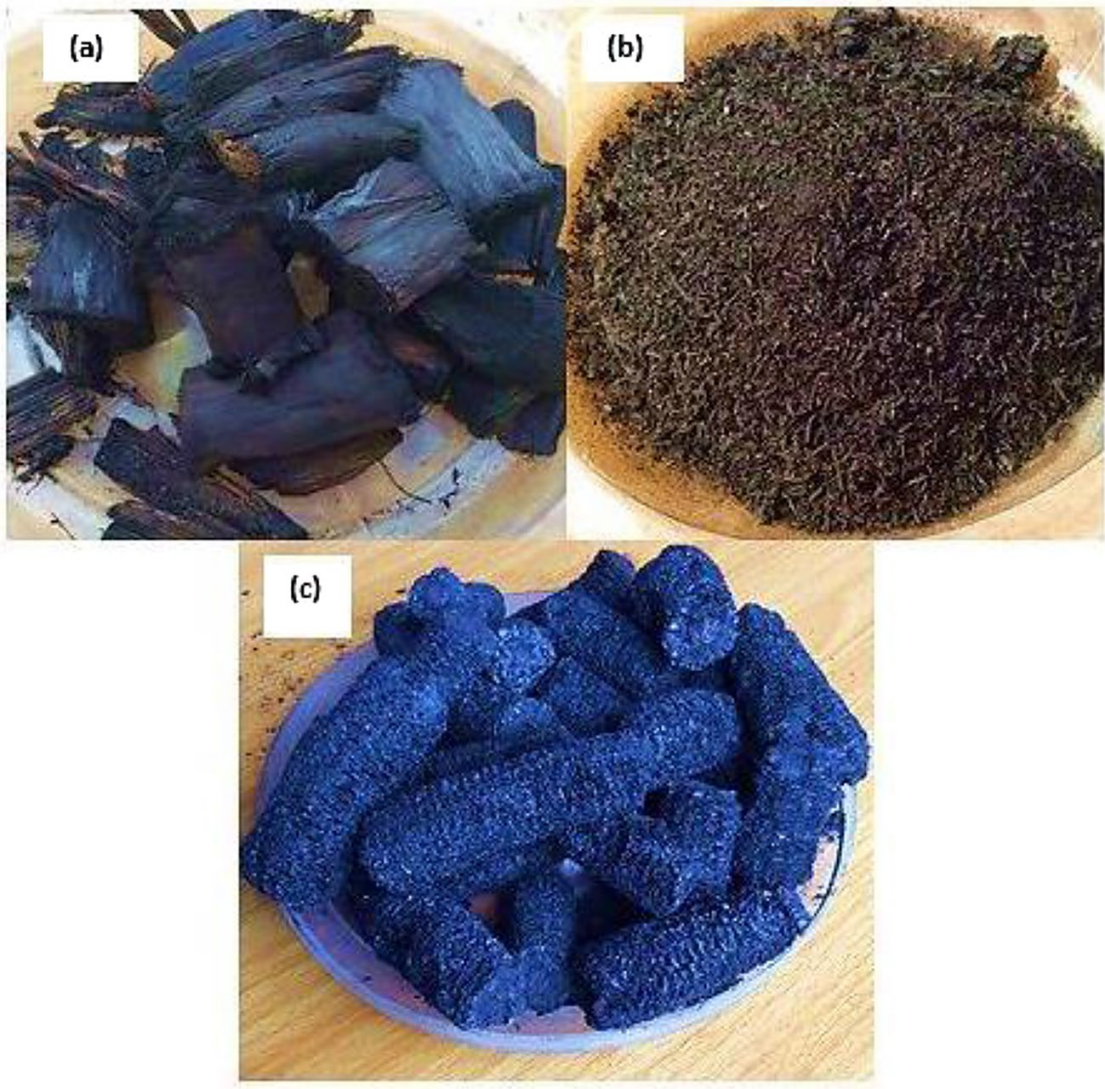
Biochar samples generated from (**a**) banana stalk, (**b**) rice husk, and (**c**) corncob. Adopted from Ibitoye et al. (2022) and modified (Open access without copyright permission requirement)

Various cutting-edge technologies are used to manufacture biochar, including pyrolysis (Mishra and Mohanty 2021), gasification (Ibitoye et al. 2021b), hydrothermal carbonization (HTC) (Ibitoye et al., 2022; Ibitoye et al. 2023b), torrefaction (Ibitoye et al. 2021c), and even innovative methods like microwave pyrolysis (Gabhane et al. 2020), and plasma pyrolysis (Bhatt et al. 2022). The viability of these methods is influenced by several parameters, including the type of feedstock, catalysts utilized, temperature, heating rates, and the configuration of the reactor (Alahakoon et al. 2022; Panwar et al. 2019; Uday et al. 2022; Zhang et al. 2022). The assessment of their effectiveness considers factors such as energy usage, product yield, and overall environmental impact (Karthik et al. 2021; Zhou et al. 2021). A detailed summary of the various biochar production methods is provided in Table 2. Each method for producing biochar has merits and demerits, making it appropriate for various uses depending on the intended product output, energy needs, feedstock accessibility, and environmental considerations. Figure 4 shows a typical biochar generated from rice, corncob, and banana stalk.

**Table 2:** Description, advantages, disadvantages, and challenges of various biochar production techniques

Biochar Production Technique	Mode of heating/ temperature	Biochar Yield (%)	Specific surface area (BET) (m²/g)	Advantages	Disadvantages /Challenges	Energy consumption	Reference
Slow Pyrolysis	-Inert/ oxygen deficient atmosphere -300-600 ^o^C	25–85	8-560	-Offers control over product yield and quality. -Generate more biochar,	-Longer processing time -Less efficient bio-oil/gas production -Energy-intensive process.	Moderate to high	(Amer et al. 2022; Nanda et al. 2016; Premchand et al. 2023a; Safarian 2023a; Safavi et al. 2023; Salimbeni et al. 2023)
Gasification	-Partial combustion of feedstock -600-1200 ^o^C	5–15	14–800	-Generate syngas for energy production. -Lower emissions	-Tar and ash issues -Lower biochar yield -Requires strict control to prevent complete combustion.	High	(Čespiva et al. 2022; X. He et al. 2021b; Qin et al. 2022; Safarian 2023a; Tauqir et al. 2019; You et al. 2017)
Hydrothermal Carbonization	-Inert/ oxygen deficient atmosphere under high pressure and water -180–260 ^o^C	30–90	2-1097	-Faster process compared to traditional pyrolysis. -Can utilize wet feedstock.	-High energy and pressure requirements. -Limited feedstock options -Biochar stability varies	Moderate	(Abhi et al. 2023; Cruz et al. 2024; Erses Yay et al. 2021; Román et al. 2020; Safarian 2023a; Shao et al. 2020; Yu et al. 2022)
Microwave and Plasma Pyrolysis	Feedstock is subjected to microwave or plasma energy. -300–800 °C. (microwave), -Plasma at a higher temperature	40–80	2-587	-Faster heating rates. -Better control over reaction conditions. -High energy efficiency	-Specialized equipment and energy requirements. -Complex process control. -Limited commercial-scale applications.	-Moderate to high -Very high (plasma)	(Akhil et al. 2021; Dermawan et al. 2022; Fodah et al. 2021; Hadiya et al. 2022; Halim et al. 2022; Kanthasamy et al. 2023; Potnuri et al. 2023; Safarian 2023a)
Torrefaction	-Inert/ oxygen deficient atmosphere -200–300 °C	50.4–89	1–80	-Produces biochar with higher energy density. -Improved grindability	-Limited carbon sequestration potential. -Challenges in mass production. -Moderate biochar yield	Moderate	(Abdelhadi et al. 2017; Chyuan et al. 2021; Govindaraju et al. 2022; Ibitoye et al. 2021b, c, 2022a, c; Safarian 2023a; Thengane et al. 2020; Yılgın et al. 2019)

**Fig. 5:**
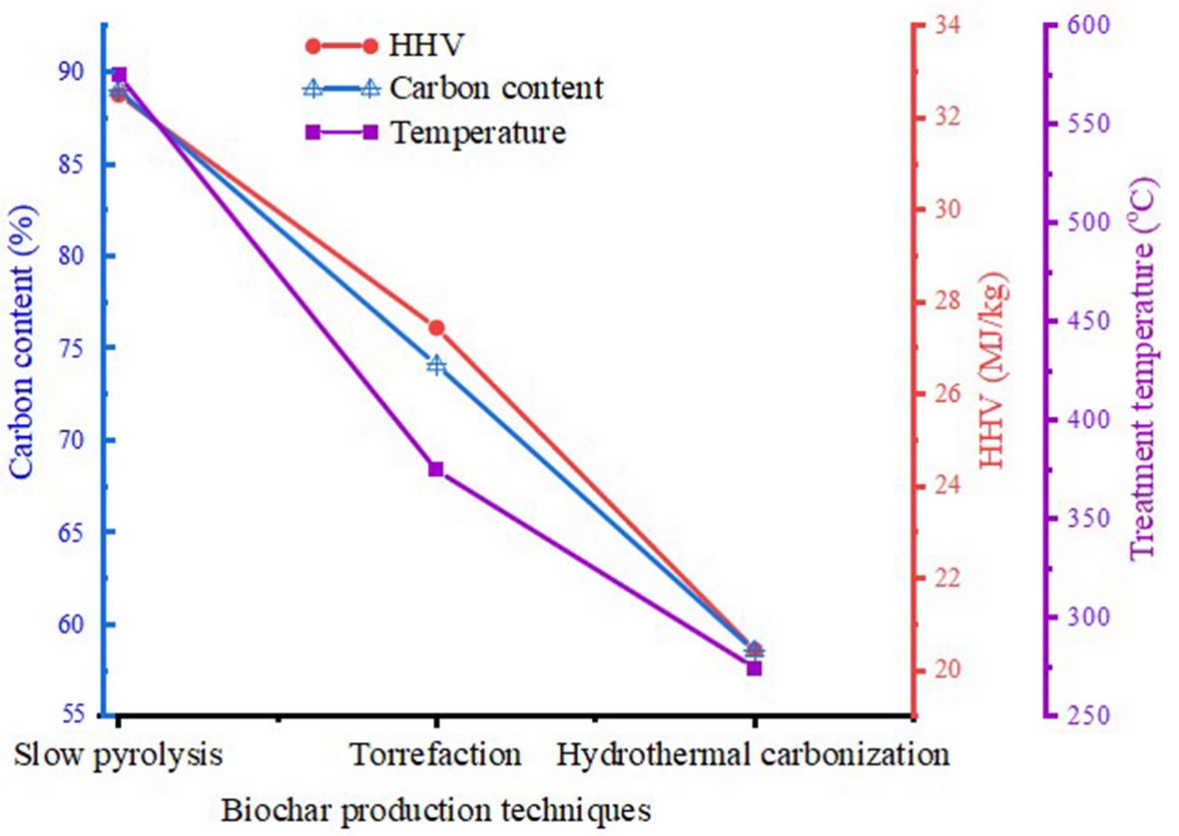
Comparison of different biochar production techniques. The data extracted from Ercan et al. (2023) and was plotted with Origin 2021 (Copyright © 2024, with permission from Elsevier)

The optimum way to produce biochar for use in steel industry applications will rely on several variables, including the demands of the ISI, the intended application of the biochar, cost, and the overall characteristics of biochar required for steel making. Analysis of the characteristics displayed in Table 2; Fig. 5 (data extracted from Ercan et al. (2023) revealed that slow pyrolysis stands out as a potential approach for producing biochar in the context of the steel industry.

Compared to other processes like fast pyrolysis and gasification, which may prioritize the generation of bio-oil or syngas, hydrothermal carbonization, and slow pyrolysis are recognized for generating larger biochar yields (Salimbeni et al. 2023). These results aligned with the reports of Abhi et al. (2023); Safarian (2023a), which revealed that slow pyrolysis and HTC are the most effective techniques for producing high-yielding biochar, with yields that vary from 25 to 90 weight%, and even more subject to the reactor type, feedstock, and operating circumstances. This finding was corroborated by another study, which showed that for each of the various applications, including recarburizing in steel-making, coal blend for coke-making, coke breeze for sintering, coal injectant for the BF, the characteristics of biochars generated through pyrolysis of biomass can be customized, enabling optimum efficiency and greater usefulness of the char (Jahanshahi et al. 2014).

The potential need for enormous quantities of biochar for iron and steel industrial use as a reducing agent is in line with this improved biochar yield. This is because of the extended dwelling time at lower temperatures during slow pyrolysis. The biochar generated is characteristically more stable and rich in carbon (Nega et al. 2023; Rathod et al. 2023). Further, the lower energy required and improved properties of biochar produced are desirable features for large-scale industrial utilizations and may reduce operating costs. It recurrently results in biochar with more structural stability and less volatile material (Premchand et al. 2023a). This can result in better biochar properties and transportation qualities, which are essential when considering integrating biochar into ISI (Liu et al. 2023; Safarian 2023b).

Studies and uses of slow pyrolysis have been made at many scales, including industrial ones (Kalderis et al. 2020; Mishra and Mohanty 2021; Safavi et al. 2023; Salimbeni et al. 2023). The technique may be scaled up, based on this experience, to satisfy anticipated demand from the steel industry.

In the summary of the report submitted by Abhi et al. (2023), it was suggested to use slow pyrolysis and a hybrid HTC method to turn biomass into biochar, which has the prospective to substitute pulverized coal injection in the BF and other petroleum-based fuels in the production of iron and steel. Furthermore, torrefaction was found unsuitable for producing high-quality biochar because of its limited ability to substantially reduce biomass AC and low heating value (Abhi et al. 2023). The conventional slow pyrolysis process for woody biomass increases ash concentration, making it unsuitable for agricultural biomass unless the ash percentage is extremely low. The suggested hybrid HTC and slow pyrolysis process initiatives address ash-related difficulties, making agricultural biomass viable for iron and steel production, notably in BF injection, which is the most common use. This hybrid approach has the potential to transform medium-ash agricultural biomass into low-ash biocarbon, improving its appropriateness for BF injection and encouraging the use of renewable feedstock.

It’s crucial to remember that the selection of the biochar production technique should be based on a careful examination of the unique requirements of the steel sector- low ash, no contaminants, consistent composition, purity, uniform particle size, and cost-effectiveness-including the planned use of biochar (Echterhof and Pfeifer 2014; Te et al. 2021; Yaashikaa et al. 2020). Additionally, ongoing studies and improvements in biochar production methods can result in additional enhancements and optimizations that could affect this evaluation.

A significant portion of the world’s steel production may be able to switch to using biochar instead of coal or coke in the coming decades (Safarian 2023a; Xia et al. 2023). The adoption of biochar from biomass as an alternative source of eco-friendly carbon for ISI will be meaningfully influenced by other factors, such as economics and technical aspects of using biochar in steel-making. Additionally, in all scenarios, including biomass sources, transportation is anticipated to be a major parameter that affects the price of biochar supply to steel industries (Norgate et al. 2012).

### Efficiency metrics in biochar production

The economic viability, environmental impact, and general practicability of biochar for various uses are all strongly influenced by how efficiently biochar is produced. Metrics for efficiency include yield, the quality of the biochar produced, and energy use (Campion et al. 2023; Zhang et al. 2022). Equation 1 offers a general representation of conversion efficiency for each biochar production process, though actual efficiency can vary based on several factors, including operating conditions, feedstock characteristics, and equipment used (Farghali et al. 2022; Premchand et al. 2023a; Sivaranjanee et al. 2023).

1 $${\rm{Conversion}}\begin{array}{*{20}{c}}{}\end{array}{\rm{efficiency}}\begin{array}{*{20}{c}}{}\end{array}\left( {\rm{\% }} \right) = {\rm{}}\frac{{{\rm{Mass}}\begin{array}{*{20}{c}}{}\end{array}{\rm{of}}\begin{array}{*{20}{c}}{}\end{array}{\rm{biochar}}\begin{array}{*{20}{c}}{}\end{array}{\rm{produced}}}}{{{\rm{Mass}}\begin{array}{*{20}{c}}{}\end{array}{\rm{of}}\begin{array}{*{20}{c}}{}\end{array}{\rm{biomass}}\begin{array}{*{20}{c}}{}\end{array}{\rm{input}}}} \times 100$$

#### Yield, quality, and energy consumption

The term “biochar yield” describes how much biochar is created from the amount of feedstock used. A high yield is preferred to increase the amount of useful biochar produced. More biochar is usually produced by comparing slower methods, like slow pyrolysis, to faster ones, like fast pyrolysis or gasification. Producing biochar has an economic component as well: yield. The physico-chemical characteristics of the biochar, such as surface area, porosity, carbon content, and stability, also impact its quality (Azzi et al. 2022; Hamidzadeh et al. 2023). Higher-quality biochar often has a higher potential for soil improvement, carbon sequestration, and industrial uses (Reddy et al. 2019; Zhang et al. 2012).

Energy useage is another important aspect of manufacturing biochar, impacting both its environmental footprint and cost (Lin et al. 2023; Patwa et al. 2022; Torres-Rojas et al. 2011). Increased energy needs may result from higher temperatures and quicker heating rates, as demonstrated in fast pyrolysis and some gasification processes. Slow pyrolysis, which occurs at lower temperatures, could require less energy but take longer.

#### Feedstock, temperature, residence time

The chemical makeup of various feedstock, such as wood, agricultural waste, or algae, varies. Feedstock composition significantly influences biochar yield, quality, and characteristics (Bhatt et al. 2022). Comprehensive characteristics of different biomass with corresponding biochar yield and quality have been reported in the literature (Safarian 2023a). High lignocellulosic biomass typically produces higher-quality biochar that is useful for various applications. The process temperature impacts the characteristics of biochar (Selvarajoo et al. 2022). Biochar with a higher FC and more excellent stability is frequently produced at higher temperatures. Slow pyrolysis generates biochar with highly developed carbon structures and increased carbon retention (Safavi et al. 2023; Salimbeni et al. 2023).

The rate of chemical reactions hinges on how long biomass residence at high temperatures. Longer residence times result in enhanced carbonization, as revealed by slow pyrolysis, resulting in well-developed and stable biochar (Uday et al. 2022).

## Technical feasibility and adaptation challenges

There are many chances for innovation and sustainability when biochar is incorporated into current industrial processes, but there are also a lot of obstacles to overcome in terms of technological preparedness, entrance hurdles, and adaptability.

Iron and steel production faces financial obstacles due to the high energy needed for biochar production and the costs associated with producing, processing, and shipping biochar (Mathieson et al. 2015). These obstacles also affect the transition from coal to bio-based fuels. Mathieson et al. (2015) emphasize the importance of establishing equivalent supply chains for the collecting, conversion, and delivery of biomass. However using charcoal made from biomass has significant potential of lowering the greenhouse gas impact of steel production.

Regulatory, logistical, and technological obstacles must be overcome to successfully integrate biochar into current iron and steel industrial processes. Technological challenges include maintaining constant biochar quality and streamlining pyrolysis technology for effective biochar production. The summary of the technological readiness, barriers to entry, and adaptation challenges of biochar use in ISI are presented in Table 3, along with the challenges of biochar production and use in ISI and possible solutions enumerated in Table 4.

**Table 3:** Technological readiness, barriers to entry and adaptation challenges of biochar use in ISI (Askeland et al. 2019; Ayaz et al. 2021; He et al. 2021a; Khanna et al. 2019; Mukherjee et al. 2022; Rodriguez et al. 2020; Sajdak et al. 2023; Yaashikaa et al. 2019; Zhou et al. 2018)

Technological readiness	Barrier to entry/ adaptation challenge
- Biochar production technology is known easily obtainable. -Industrial use of biochar in iron and steel is developing. - Continuous sensitization and awareness on large-scale adoption effective operation. - Ongoing research on the optimization biochar properties for iron and steel industrial applications. - Partial replacement of coke breeze with biochar in the sintering process shows potential for reducing GHG emissions - Biochar can be injected directly into blast furnaces as a partial substitute for pulverized coal. - Biochar can be used as a carbon source in EAFs - Potential use of biochar as a reductant in DRI processes - Growing interest in sustainable and low-carbon technologies among industry stakeholders	- High price of biochar compared to fossil-based carbons - Transport and supply issues - Integration into existing production process requires optimization and modification. -Sustainability of efficiency or quality of steel production - Varying feedstock properties - Pyrolysis conditions and biochar properties require standardization - Weak feedstock supply networks - Adjusting operational factors (temperature, pressure) to incorporate biochar without compromising production. - Proper handling, storage, and safety procedures are crucial for maintaining biochar quality and safety. - Training for engineers and laborers in biochar use and integration. - Managing emissions and byproducts to comply with environmental regulations.

**Table 4:** Regulatory, logistical, and technological challenges of biochar production and use in ISI and possible solutions (Bach et al. 2016; Jayamini et al. 2024; Khanna et al. 2019; Ko et al. 2018; Ladu and Vrins 2019; Mousa et al. 2016; Vereš et al. 2014; Ye et al. 2019)

Challenge	Solution	Recommendation
**Technological Hurdles**		
1. Inefficiency of current pyrolysis technologies for large-scale biochar production	- Invest in advanced pyrolysis technologies.	- Targeted research and development on improving pyrolysis efficiency.
	- Continuous improvement through R&D and pilot projects.	- Provide funding and grants for biochar-related research.
2. Variability in biochar quality due to differences in feedstock and pyrolysis conditions	- Develop standardized protocols for biochar production. - Certification programs and quality assurance mechanisms.	- Establish industry standards for biochar quality control. - Collaborate with regulatory bodies to develop consistent quality standards.
3. Compatibility of biochar with existing industrial processes	- Collaborative R&D efforts between biochar producers and industrial users.	- Public-private partnerships to pool resources and expertise.
	- Customize biochar properties to meet industrial process requirements.	- Launch pilot projects to demonstrate feasibility and benefits.
**Logistical Hurdles**		
1. Non-reliable and inconsistent supply of biomass feedstock	- Develop regional biomass supply chains.	- Establish biomass collection centers and contracts with suppliers.
	- Implement efficient transportation systems.	- Invest in biomass transportation infrastructure.
2. Transportation and storage of biochar and biomass challenges	- Design dry, sealed containers for biochar storage.	- Utilize existing logistics networks within the iron and steel industries.
	- Prevent contamination and moisture uptake during transport.	- Implement best practices for biochar handling and storage.
**Regulatory Hurdles**		
1. Inconsistent regulatory frameworks	- Advocate for harmonization of regulations.	- Engage with policymakers to promote the benefits of biochar.
	- Develop industry standards in collaboration with regulatory bodies.	- Collaborate on the development of consistent regulations and incentives.
2. Challenges in ensuring that biochar production and use comply with environmental and safety regulations	- Conduct comprehensive environmental impact assessments. - Implement monitoring and reporting mechanisms.	- Establish stringent safety protocols for biochar handling. - Conduct regular audits to ensure compliance with environmental standards.

### Case studies on biochar utilization

Some case studies on biochar utilization in ISI focusing on foaming characteristics, reactivity, reducing agent and injection potentials, sintering, and CO_2_ reduction characteristics were discussed in this section.

Ensuring a consistent biochar supply poses a challenge due to the enormous raw material requirements of the ISI. For instance, Brazil, with abundant biomass resources, has historically produced high-quality iron and steel using biochar from woody biomass due to limited coking coal reserves. Currently, Brazil leads in industrial biomass and biochar use for steelmaking, primarily in mini blast furnaces, leveraging its status as the largest wood-based biochar producer (Mandova et al. 2018). However, labor costs, forest regeneration cycles, and environmental regulations still affect consistent biochar production (Feliciano-bruzual 2014).

#### Biochar foaming reactivity characteristics

**Fig. 6:**
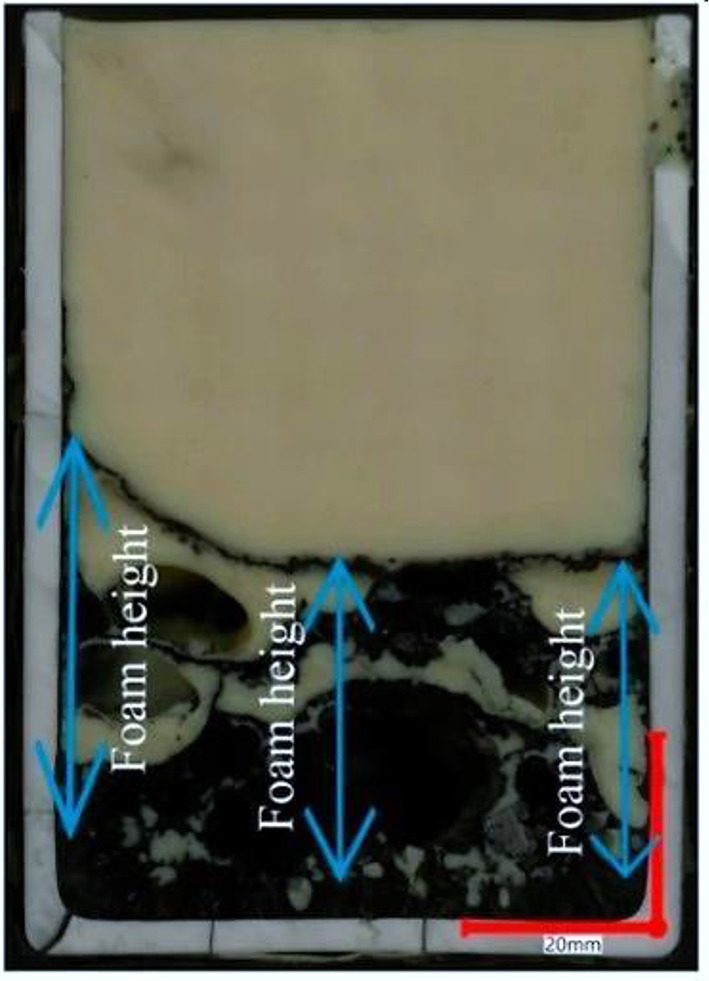
Foaming characteristics and measurement. Adopted from Kieush and Schenk (2023) (Open access without copyright permission requirement)

Preliminary laboratory tests have been conducted to compare the foaming behavior of biochar and coal. The study used slag samples from the EAF in alumina crucibles filled with pulverized biochar or coal and heated to 1600 °C (Bianco et al. 2013). Biochar produced at 400, 500, and 600 °C was used for the trials. The foaming tendency was assessed by measuring the height of the foam generated in the crucible (Fig. 6). After the trials, analysis of the crucibles indicated that biochars produced at 400–500 °C exhibited foaming capability comparable to standard coals, suggesting their suitability for iron and steel applications (Bianco et al. 2013).

The biochars generated at 500 °C and 600 °C exhibited reactivity levels similar to standard coal. The 500 °C temperature is considered a suitable compromise for pyrolysis because it optimizes the heating value of char and syngas.

#### Biochar as an alternative to coke breeze

**Fig. 7:**
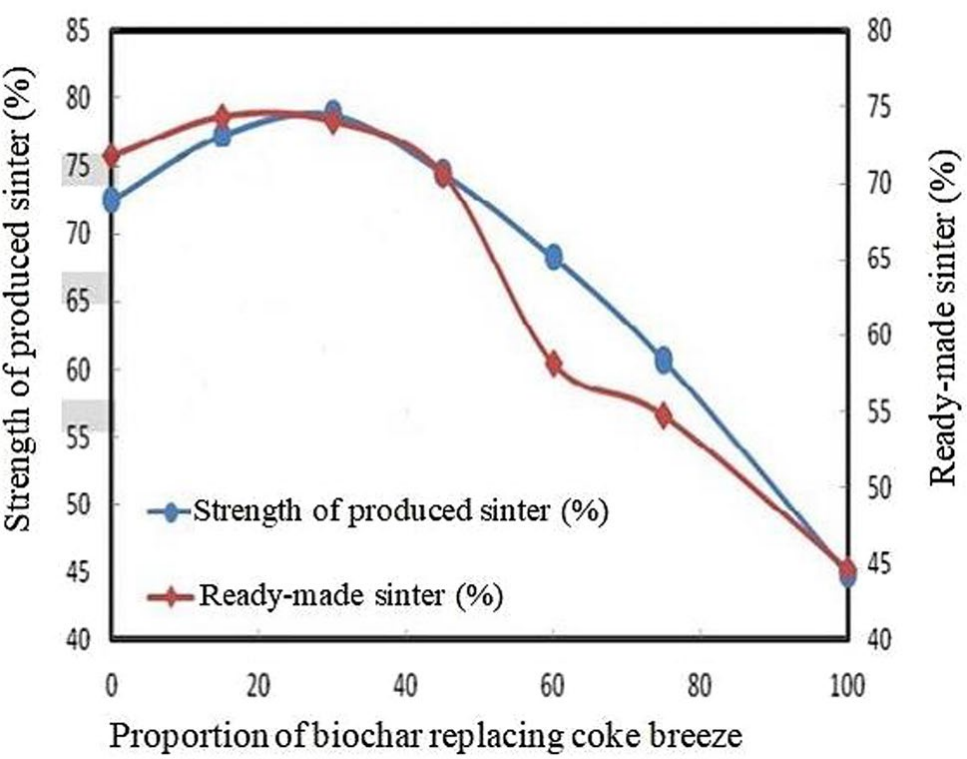
Effect of replacing coke breeze with biochar on the strength and volume of the resulting sinter. Reprinted from El-Hussiny et al. (2015) (Open access without copyright permission requirement)

**Fig. 8:**
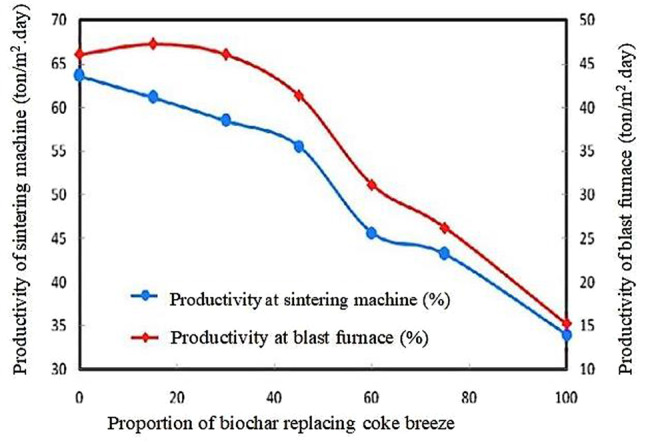
Effect of biochar utilization as a substitute to coke breeze on the performance of sintering machine and BF. Reprinted from El-Hussiny et al. (2015) (Open access without copyright permission requirement)

Figures 7 and 8 depict how replacing coke breeze with biochar affects the volume and strength of the resulting sinter (El-Hussiny et al. 2015). Figure 7 shows that as the percentage of biochar replacement increases, the quantity and strength of the produced sinter increase, reaching their peak at 30% biochar replacement. However, going beyond 30% biochar replacement has a negative impact on both the strength and quantity of the sinter, attributed to the faster combustion of biochar compared to coke breeze. As shown in Fig. 8, the optimal productivity levels for the sintering machine and the blast furnace yard were achieved by replacing 30% of coke breeze with biochar in the sinter raw mix, resulting in approximately 59% and 46%, respectively. This outcome was attributed to the reduced sintering time. It suggests replacing coke with biochar could enhance sinter productivity and maintain a reasonable yield (El-Hussiny et al. 2015).

#### Potential of biochar for reduction of greenhouse gases

**Fig. 9:**
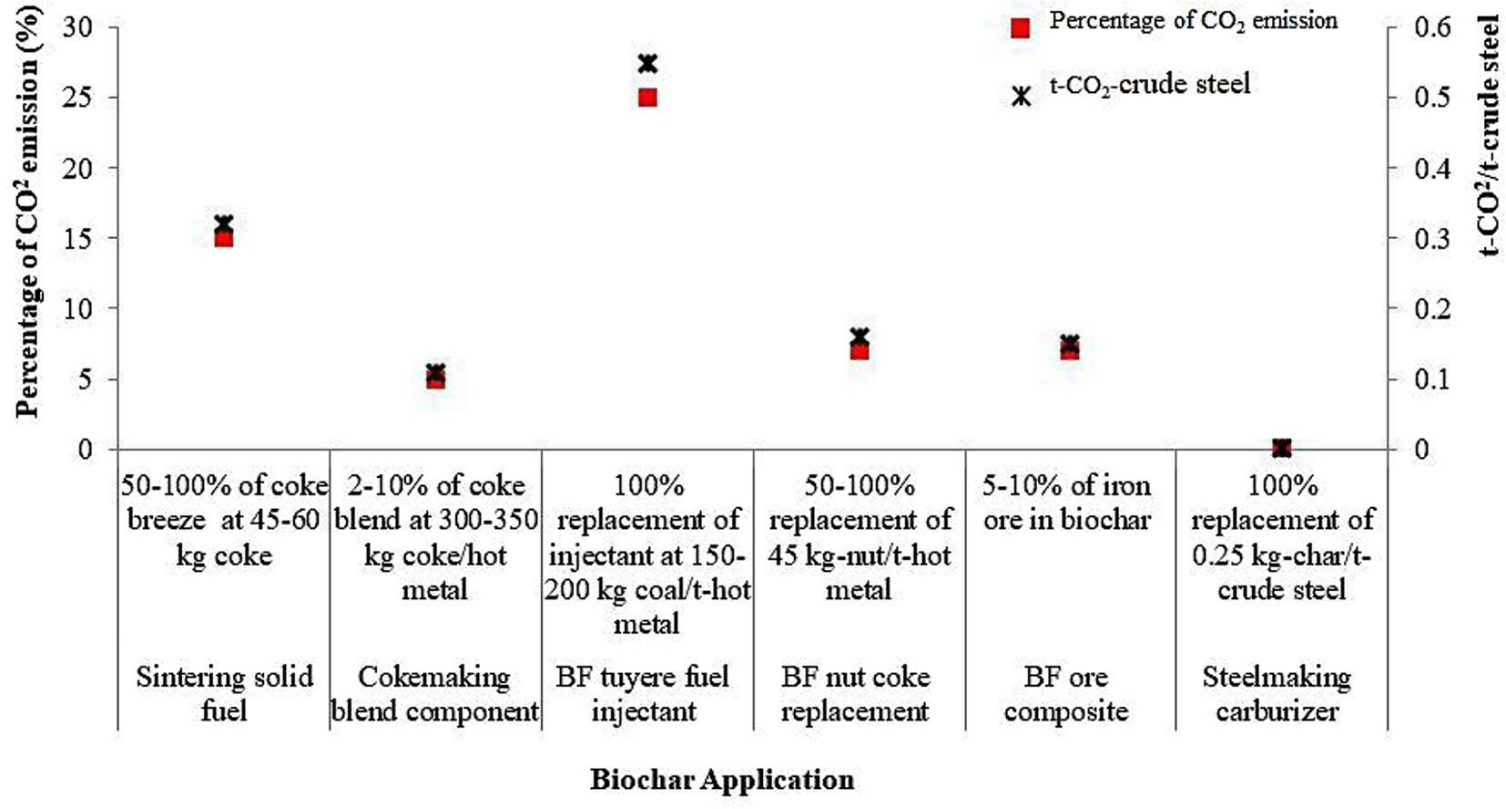
Environmental implication of biochar utilization in an integrated steel-making process. The data was extracted from Mathieson et al. (2015) and was plotted with Microsoft Excel 2023 (Copyright © 2024, with permission from Elsevier)

Australia’s major steel companies, Arrium and BlueScope Steel, have teamed up with the Commonwealth Scientific and Industrial Research Organisation (CSIRO) for the Australian Steel Industry CO_2_ Breakthrough Program, leading to the development of two key technologies: biochars in ironmaking and dry slag granulation with heat recovery (Jahanshahi et al. 2013; Pandit et al. 2020). Successful lab trials of the technologies have shown promising CO_2_ emission reductions. By replacing coal-based coke with biochar, low-carbon steel production becomes feasible. Australia’s resources could produce 1 Mt/y of biochar at costs comparable to coal/coke (Jahanshahi et al. 2013; Pandit et al. 2020). Figure 9 outlines applications of biochar in the Australian integrated steelmaking process. Significant CO_2_ emission reductions (32–58%) are possible, mainly through BF tuyere injections. However, Australia’s abundant coking coal and low cost compared to biochar present challenges.

Replacing coal/coke with biochar in BF iron-making offers several advantages, including reduced CO_2_ and SO_2_ emissions, potential reduction in slag quantity, and improved hot metal quality (Fig. 9). Studies have indicated that utilizing biochar with specific characteristics such as VM less than 10%, particle size smaller than 1 mm, and density greater than 700 kg/m^3^ can lead to CO_2_ emissions savings ranging from 1 to 5% in coke making (Jahanshahi et al. 2013; Pandit et al. 2020). Similarly, in replacing BF nut coke, biochar with VM less than 7%, high density, and particle sizes ranging from 20 to 25 mm can result in estimated CO_2_ emissions savings of 3–7%. In iron ore sintering, the utilization of biochar, especially with low VM, high densities exceeding 700 kg/m^3^, and small sizes ranging from 0.3 to 3 mm, can lead to CO_2_ reductions of 5–15%. Furthermore, when used as BF injectant, biochars with VM between 10 and 20%, AC less than 5%, and low alkali levels have the potential to yield substantial CO_2_ emissions savings of 19–25% (Jahanshahi et al. 2013; Pandit et al. 2020).

**Fig. 10:**
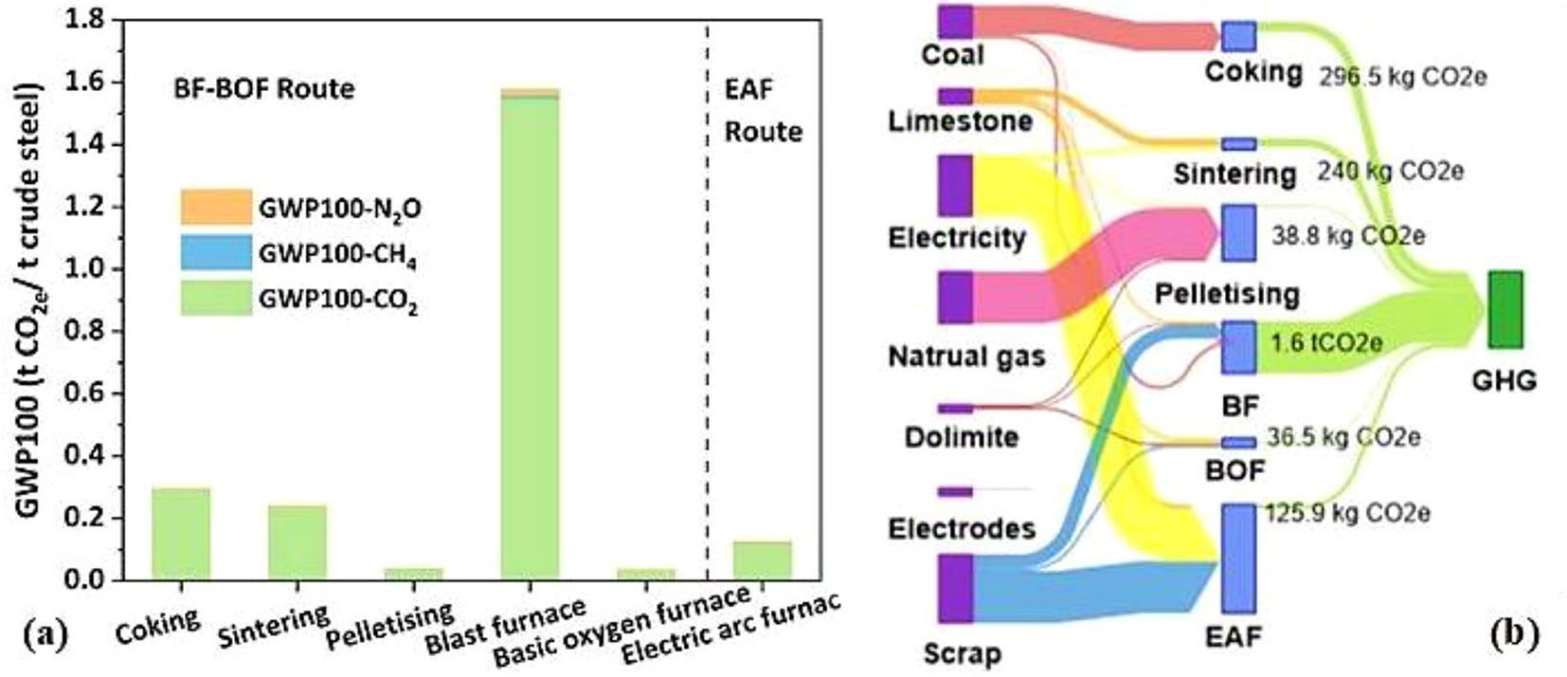
Raw material flow and greenhouse gas emissions in a typical ISI. Reprinted from Meng et al. (2024) (Copyright © 2024, with permission from Elsevier)

Meng et al. (2024) studied the carbon emissions of an iron and steel plant. The report showed that the BF-BOF route emitted more global warming potential (GWP100) than the EAF route, with 1 ton of crude steel producing 2 tons of CO_2_e for BF-BOF compared to 0.1 tons for EAF (Fig. 10). Higher emissions in the BF-BOF route stemmed from more processes and material inputs, especially self-produced coke. The EAF route, mainly powered by electricity, had lower total GHG emissions despite higher indirect emissions from electricity use (6 kg CO_2_e per ton of crude steel) compared to BF-BOF (1 kg CO_2_e per ton). BF-BOF route’s emissions were dominated by the blast furnace (72% of GWP100), with CO_2_ being the major GHG (99%), largely due to fossil fuel consumption (2 tons CO_2_e from coke and coal). The BF required 394 kg of coke per ton of crude steel while pelletizing and sintering needed significantly less. Despite the emissions, coke, and coal are essential in iron and steel production. For long-term low-carbon transitions, Meng et al. (2024) recommended promoting the EAF route.

**Fig. 11:**
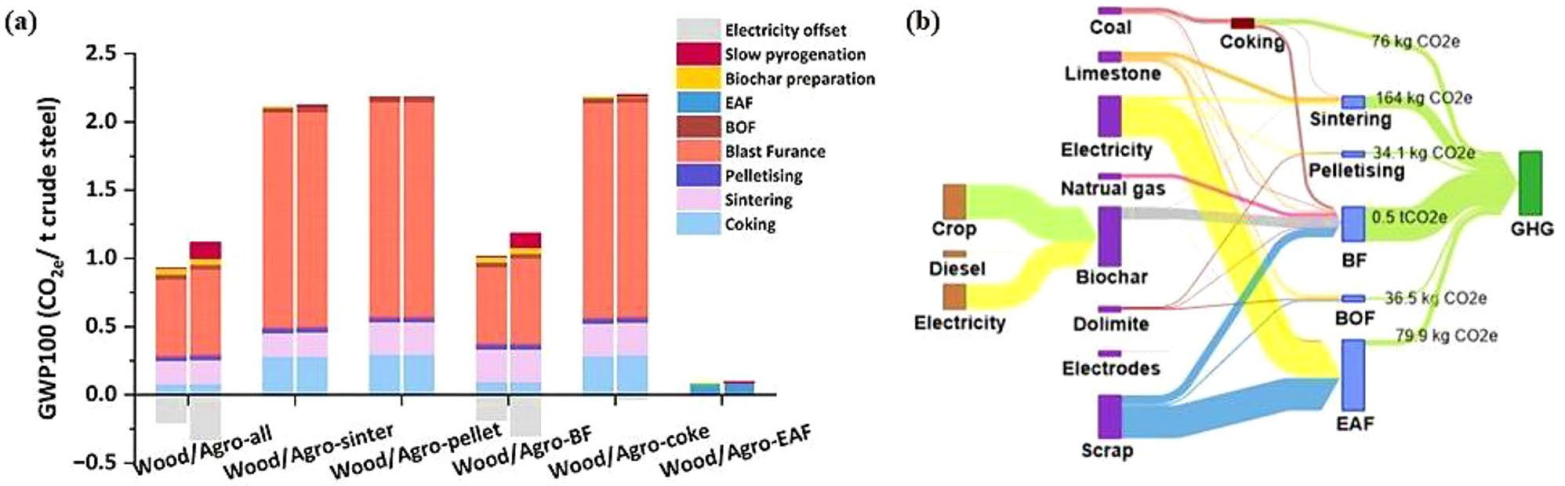
Greenhouse gas emissions in the twelve biochar substitution situations in ISI: (**a**) GWP100 emissions and (**b**) Sankey chart. Reprinted from Meng et al. (2024) (Copyright © 2024, with permission from Elsevier)

Different types of biochar exhibit varying capacities for mitigating emissions. Figure 11a and b illustrate the emissions reduction potential of individual processes using wood and agro biochars. Consistent with previous studies, replacing 6% of coke with biochar in iron and steel production can reduce CO_2_ emissions by 43% (Meng et al. 2024).

According to Fig. 11, GWP100 and CO_2_ emissions showed similar trends, with CH_4_ and N_2_O having minor impact on GWP100. However, straw-based biochar had higher GWP100 emissions (0.4 t CO_2_e/t biochar) compared to wood-based biochar (0.1 t CO_2_e/t biochar). Despite generating about 1 MWh more electricity due to higher bio-gas yield, straw-based biochar’s carbon offsets were less effective. Using modern biochar production equipment that collects and reflow bio-gas for heating can enhance carbon credits, favoring wood-based biochar, which achieves an emission reduction of about − 0.5 t CO_2_e/t biochar versus straw-based biochar’s − 0.5 t CO_2_e/t biochar.

Wood-based biochar has a higher carbon density due to its lignin structure, resulting in more efficient biochar production. Consequently, it provides greater emission reduction across all processes in both iron and steel production routes compared to straw-based biochar.

**Fig. 12:**
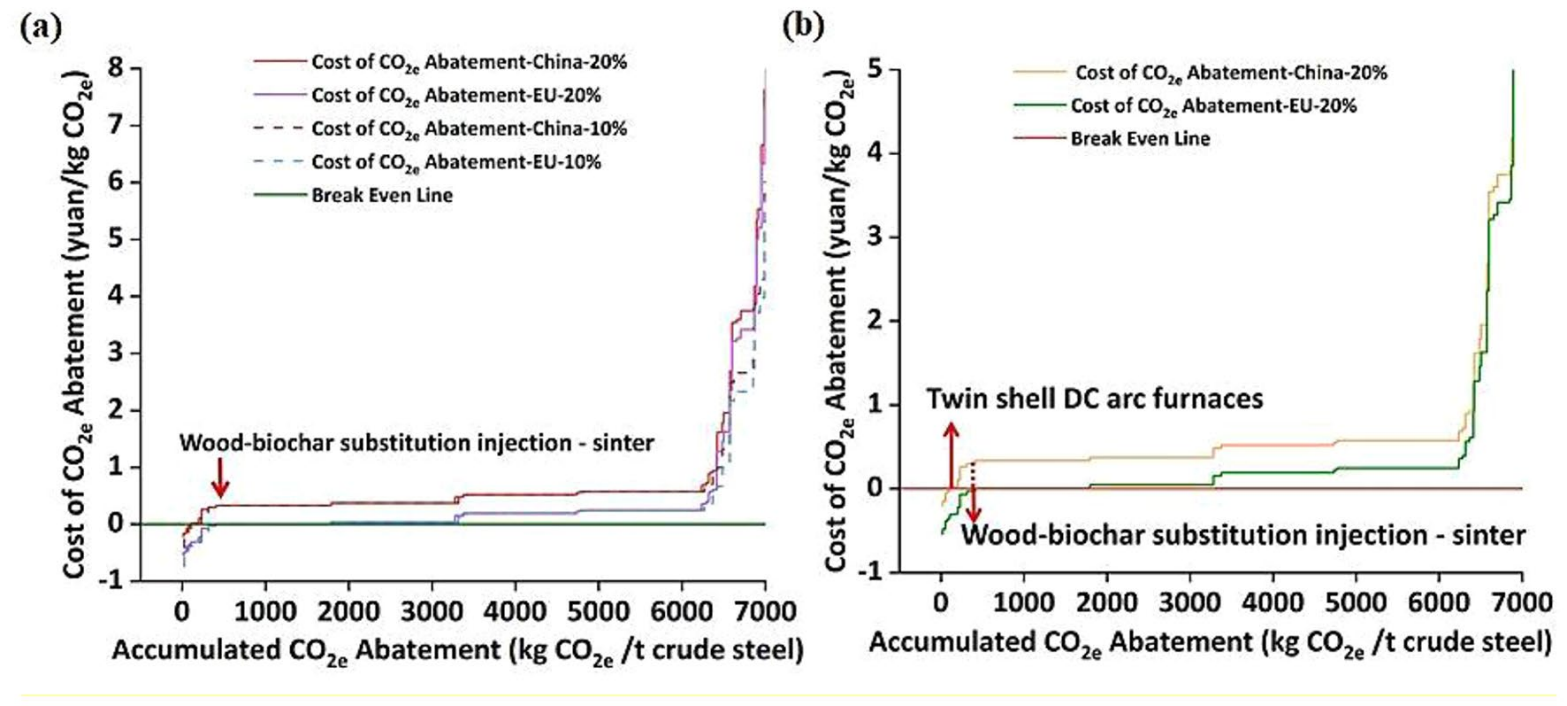
CO_2_ supply curve (SCS) of the emission reduction technologies of ISI: (**a**) CSC with a discount rate of 10% and 20% and (**b**) CSC with biochar utilization. Reprinted from Meng et al. (2024) (Copyright © 2024, with permission from Elsevier)

Meng et al. (2024) further analyzed carbon emission trading prices in China and the EU emissions trading system (ETS). In 2021, China’s average price was 0.04 yuan/kg CO_2_e, much lower than the EU ETS price of 0.4 yuan/kg CO_2_e. Biochar is more expensive than coal and coke, discouraging its use in iron and steel industries. Steel-used coke and coal cost about 1322 yuan/t in China, while wood-based and straw-based biochar cost 3500 yuan/t and 3787 yuan/t, respectively. Figure 12a and b show that without the ETS, biochar substitution lacked economic advantages. With China’s ETS, six emission reduction methods showed economic benefits, but no biochar scenarios were included. Under the EU ETS, 12 technologies, including two biochar substitution scenarios, were economically beneficial due to stricter regulation and higher carbon prices. According to the report, wood-based biochar performed better economically than straw-based biochar due to lower cost and higher GHG reduction. Biochar substitution in coking was economically unfeasible. Wood-based biochar in sintering was the most economical, potentially reducing CO_2_e emissions by 2.01 million tons in 2021.

#### Biochar reduction property

**Fig. 13:**
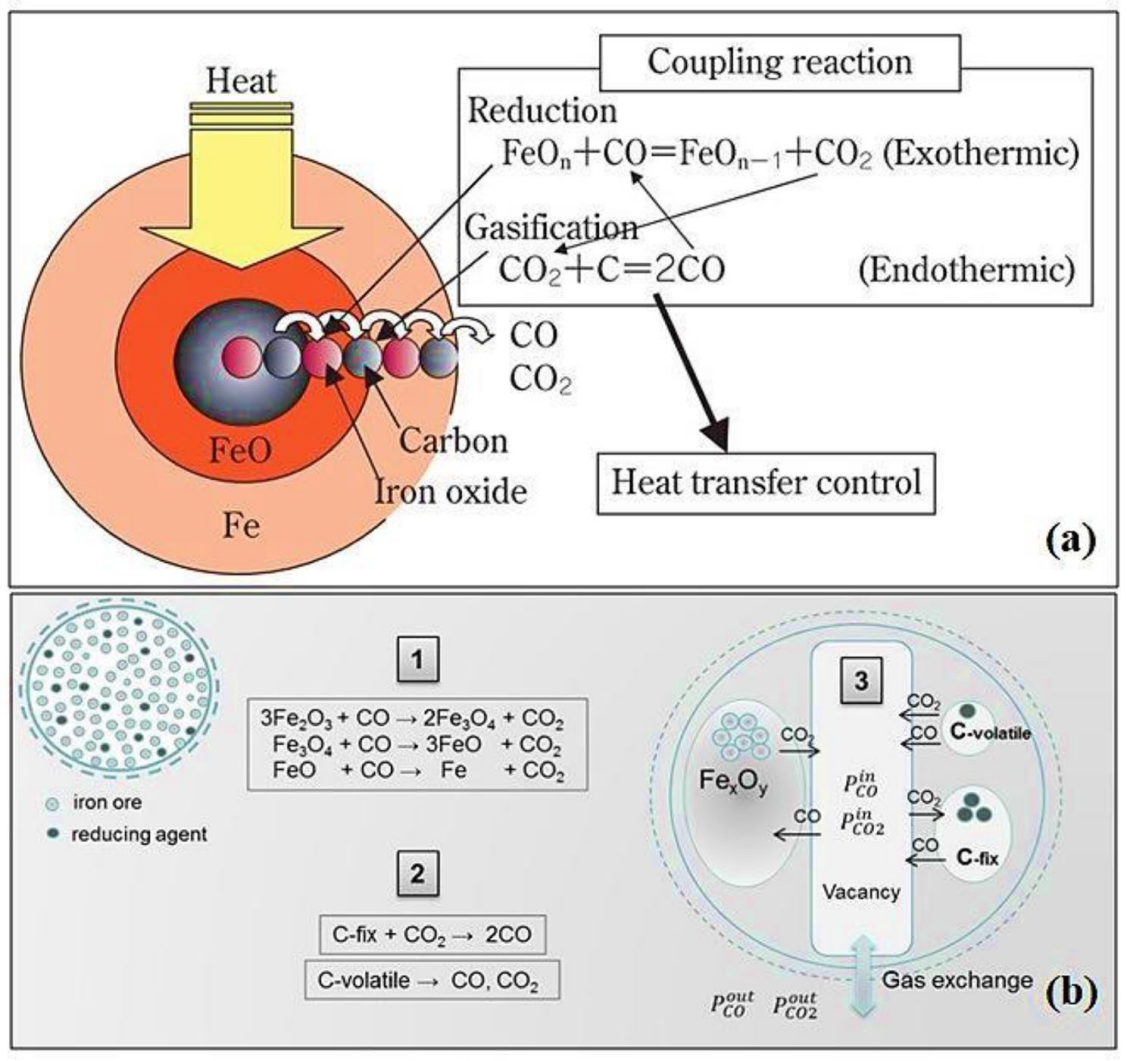
Reduction characteristics of biochar: (**a**) reduction mechanism and (**b**) reaction kinetics. Adopted from Kowitwarangkul et al. (2014) and Michishita and Tanaka (2010) (Open access without copyright permission requirement)

In iron-making, biochar can act as a reducing agent, initiating a series of reactions with iron oxides like hematite, magnetite, and Wustite (Hossein et al. 2023). This reaction cascade starts with biochar particles reacting with solid hematite (Eq. 2), followed by CO gas production (Eq. 3) and its reaction with magnetite (Eq. 4) and Wustite (Eq. 5), generating CO_2_ for the pivotal carbon solution loss reaction (Hossein et al. 2023; Kowitwarangkul et al. 2014). Additional reactions, including CO regeneration (Eq. 6), contribute to the reduction process. Moreover, biochar can yield syngas (CO + H_2_) (Eq. 7) during reduction, while residual carbon combustion produces CO_2_ (Eq. 8), offering valuable byproducts. Syngas are versatile fuel or feedstock, enhancing energy efficiency and operational sustainability (Hossein et al. 2023; Michishita and Tanaka 2010). The reduction mechanism and reaction kinetics of biochar is presented in Fig. 13.

2 $$3{Fe}_{2}{O}_{3}\left(s\right) + C\left(s\right) \to 2{Fe}_{3}{O}_{4}\left(s\right) + CO\left(g\right)$$

3 $$3{Fe}_{2}{O}_{3}\left(s\right) + CO\left(g\right) \to 2{Fe}_{3}{O}_{4}\left(s\right) + {CO}_{2}\left(g\right)$$

4 $${Fe}_{3}{O}_{4}\left(s\right) + CO\left(g\right) \to 3FeO\left(s\right) + {CO}_{2}\left(g\right)$$

5 $$FeO\left(s\right) + CO\left(g\right) \to Fe\left(s\right) + {CO}_{2}\left(g\right)$$

6 $$C\left(s\right) + {CO}_{2}\left(g\right) \to 2CO\left(g\right)$$

7 $$C + {H}_{2}O \to CO + {H}_{2}$$

8 $$C + {O}_{2} \to {CO}_{2}$$

Sahoo et al. (2022) delved into the influence of temperature, dwelling time, and various reductants on iron ore. Their findings revealed that higher temperatures and prolonged reduction time increased the degree of reduction. The research used wood dust, coconut shells, and sugar cane biochar. Through statistical analysis, they formulated a model showcasing the direct effect of temperature dwelling time on the reduction characteristics of the produced biochar. Remarkably, the FC content of the reductants showed no noteworthy influence on reduction. According to Sahoo et al. (2022), coconut shell char exhibited the highest reduction rate at 1000 °C for 60 min among the three biochars. This study underscores the potential of biochars as viable alternatives to coke within the iron and steel industries.

Here is a comprehensive study of the technical viability and adaptation challenges of biochar in ISI:

i. Interface with existing iron and steel production processes: The primary challenge is guaranteeing that the integration of biochar in the steel-making process is consistent with traditional steel production methods (Abhi et al. 2023). Different operating conditions, heating rates, temperature ranges, and materials specifications exist for various steel-making processes- blast furnaces, electric arc furnaces, and direct reduction steel-making. Biochar’s chemical and physical properties must suit these methods to ensure a smooth integration. For instance, the gasification potential, combustion characteristics, and reactivity of biochar must be examined in relation to the requirements of each operation (Qin et al. 2022).
ii. Cost associated with biochar utilization: The commercial viability of biochar generation is hindered by costs related to biomass collection, feedstock handling, transport, drying, etc., rendering biochar products less competitive compared to coal. A report has highlighted that implementing a carbon tax will be crucial in alleviating costs associated with biomass adoption in the ISI (Mousa et al. 2016a). The tax is typically applied to coal combustion, crude oil, etc., to encourage the transition to cleaner and more sustainable energy sources.
iii. Lower CSR and higher CRI: The vital high-temperature characteristics of coke, essential for sizeable modern BFs, are commonly assessed through CSR and CRI values. Coke with high CSR (above 460%) and low CRI (below 23%) is preferred for optimal performance. These properties enhance penetrability in the upper part of the shaft and improve combustion, demonstrating the necessity of high-strength coke to prevent degradation and maintain permeability in the BF skeleton (Mousa et al. 2016a).
iv. Ash-related challenges: The AC significantly affects the calorific value of biochar and the heat balance and slag-forming reactions in the BF. Different biomass sources have unique ash compositions; for instance, agricultural biomass often contains K_2_O and SiO_2_, whereas a higher CaO content characterizes woody biomass. Effectively managing components like zinc, lead, alkalis, phosphorus, and sulfur is vital in the BF to prevent operational challenges and ensure steel quality, with sulfur and phosphorus posing specific risks (Abhi et al. 2023). Therefore, addressing ash-related concerns is crucial for the seamless integration and implementation of the process.
v. Integration and process modifications: It may be essential to make significant technological changes to adapt steel manufacturing processes to accommodate biochar production and consumption. Rebuilding gear, improving temperature profiles, establishing biochar manufacturing units, and ensuring proper handling practices are just a few of the challenges that may occur. Management of biochar feed rates, distribution, and burning may necessitate the development of new apparatus or control systems.
vi. Emission monitoring: The potential for CO to be produced by biochar during heating or combustion needs to be appropriately controlled. It is crucial to comprehend the CO generation capability of biochar and how it interacts with the steel-making reactions in processes where CO is a valuable reducing agent. The optimal use of CO must be achieved while averting unfavorable results. Hence, appropriate control methods must be in place.
vii. Residue management: Biochar production often leads to the generation of byproducts, including ash and VM (Lin et al. 2023; Premchand et al. 2023a). It is vital to manage and dispose of these byproducts in an environmentally-friendly manner. The complete process design must include techniques for collecting VMs or gases, treating the resulting ash, and eliminating possible emissions.
viii. Necessities for research and development (R&D): Continuous R&D efforts are required to solve technical issues. The suitability of biochar characteristics in iron and steel production should be studied in detail, including its impact on product quality, emissions, and process optimization. To develop novel solutions, it is essential that experts in process engineering, biochar technology, and iron and steel manufacturing work together.
ix. Operational efficiency: Any variations to current iron and steel manufacturing methods may impact operational efficiency. Evaluating the possible effects of biochar integration on process and energy efficiency and product quality is vital. Cautious planning is necessary to minimize process disruptions and avoid unexpected consequences such as increased energy consumption, product output, and quality reduction.

## Cost implications, scalability, and long-term sustainability impacts of biochar production and applications in ISI

The cost implications like start-up costs, recurring costs, possible cost savings, and financial incentives like tax breaks and subsidies impact the biochar production and applications in ISI. Variables that affect the scalability and long-term sustainability of biochar utilization are discussed in this section.

### Cost implications of biochar production and application in ISI

Start-up cost: The design and construction of a biomass conversion plant, such as pyrolysis setup and other biochar production technologies and processes to establish biochar production facilities that can match ISI demand are capital intensive (Alias et al. 2014). The initial cost of modifying the existing ISI facilities is another significant expense (Gu et al. 2023). This can entail making modifications to feedstock handling systems, furnaces, and storage facilities. Nonetheless, these costs might be lessened using an integrated approach to cost reduction in steel production planning, especially in marginally profitable operations (Pelser et al. 2022). Furthermore, research and development cost implications are necessary to develop and optimize the biochar production technique that is appropriate for large-scale industrial uses (de Jong et al. 2017; Purohit et al. 2018).

Operational costs: The biomass availability and cost of collection depend on factors including location, season, and competing applications (Berry and Sessions 2018). For instance, the cost of transportation of biomass feedstock from collection site to biochar production facility, and transporting biochar to iron and steel plants contribute to the overall operational cost of the ISI (Berry and Sessions 2018). These costs can be substantial, especially when the feedstock collection location, biomass production facilities, and iron and steel plants are far from one another (Cheng et al. 2020b). The energy required for biochar production contributes to the industrial operational costs. In addition, the control of biomass feedstock supply chains, running and maintaining biochar production facilities, and integration of biochar into iron and steel industry processes require professional personnel. To ensure continuing and efficient output, industrial equipment has to be constantly monitored and maintained. This is especially crucial given the competitive nature of the global iron and steel market and the requirement for effective manufacturing methods. These actions can greatly raise the entire cost of maintenance, repairs, and part replacement.

Cost savings: Biochar has the prospect of earning carbon credits under several carbon trading programs because it minimizes the emission of greenhouse gases (Salma et al. 2024). The organization can generate revenue by selling these credits or using them to offset carbon taxes. In addition, an organization may escape sanctions and improve its corporate sustainability reputation by using biochar as an alternative to traditional carbon, thus reducing emissions and complying with environmental regulations (Salma et al. 2024). Furthermore, the conversion of industrial wastes into biochar reduces the cost of disposing of industrial wastes and residues (Ghosh et al. 2023; Gunarathne et al. 2019). The wastes are converted into useful products to transport and processed at the landfills.

Governmental support: Governments and financial institutions may provide subsidies, grants, or low-interest loans to encourage the adoption and implementation of sustainable practices like biochar production as an alternative to fossil fuels. The start-up and operational costs of biochar production and implementation in ISI can be reduced considerably via governmental support. Bach et al. (2016) and Vochozka et al. (2016) raise concerns about the economic obstacles to widespread biochar use, including the lack of compelling evidence for yield increases and the need for financial incentives. Another study suggests that government policy support, including financial incentives, nonfinancial policy support, and research and development funding, can play a crucial role in driving commercial-scale biochar production and use (Pourhashem et al. 2019). Similarly, business owners participating in sustainable practices and technology can benefit from tax incentives, which lower their overall tax burden (Dinis et al. 2023; Lind 2021). For example, Portugal’s tax incentive for digital transformation positively correlates with companies’ financial performance (Dinis et al. 2023).

### Scalability of biochar production and application in ISI

The factors that impact the scalability of biochar production and use in ISI include feedstock availability, technology advancement and innovations, market demand, and compatibility with the existing industrial processes. The main sources of biomass feedstock for biochar production are forest residues, agro-waste, municipal solid wastes, and energy crops (Ibitoye et al. 2023b). The availability of feedstock and the financial feasibility of large-scale production continue to be major obstacles despite the potential advantages of biochar in agriculture, such as soil improvement and carbon sequestration (Nair et al. 2017). Season and location can have an impact on feedstock availability, which is necessary for the scalability of biochar synthesis and utilization (Phillips et al. 2018). For example, agricultural residues are available in huge amounts in an area with large forests and intensive agricultural activities, especially during the harvest period. Another essential component for collecting and transporting biomass feedstock to biochar manufacturing plants is an effective logistics and transportation network. Mapping and identifying biomass resources through regional evaluations might aid in feedstock supply chain optimization (Hogland et al. 2018).

The scalability of biochar production and applications in ISI requires the utilization of advanced technologies, which are efficient, and able to handle a variety of biomass. These include but are not limited to advanced automation design engineering, and process control. In their respective works, Kumari et al. (2023) and Rex et al. (2023) emphasize the significance of temperature, heating rate, and feedstock type in the creation of biochar. Rex et al. (2023) examined the application of machine learning techniques to enhance the process. The combined findings of these studies highlight the potential of cutting-edge technology, including automation, design engineering, and process control, to enhance the yield and efficiency of biochar. Furthermore, it is crucial to adopt simple and affordable biochar production technology to optimize biochar generation with minimized costs and energy. Moreover, it is vital to ensure that the implementation of biochar advanced technologies in ISI biochar works with the existing iron and steel production process, such as use in blast furnaces without sacrificing product quality or efficiency.

The adoption of biochar on a larger scale in ISI depends on the ability of the biochar producer to manufacture biochar comparable with coal and coke (creation of value-added products). This involves setting and maintaining biochar of high-quality standards, and sensitizing ISI to the benefit of biochar utilization are necessary to create a stable market and demand for biochar by iron and steel producers (Ye et al. 2019). Wider adoption of biochar in ISI may be accelerated by showcasing the economic and environmental benefits of biochar. The creation of policies and regulations such as carbon pricing schemes, renewable energy requirements, and sustainability standards can encourage the ISI and other domestic and industrial stakeholders to adopt the use of biochar (Pourhashem et al. 2019). Large-scale production and application can also be facilitated by prompt delivery of biochar to ISI, and feedstock to biochar production facilities, which can be achieved by effective supply chain management (Anderson et al. 2016).

### Long-term sustainability impacts of biochar production and application in ISI

The long-term sustainability impacts of biochar production and use in ISI are related to their environmental, economic, and social consequences. Carbon may be stored in biochar for thousands of years (Kamali et al. 2022). The application of biochar in ISI can greatly lower the carbon footprint of the sector, which complies with the United Nations Sustainable Development Goals (SDGs) to mitigate climate change (Gebara and Laurent 2023; UN 2019). The use of biochar as an alternative to fossil fuels and other traditional carbon-intensive sources can help minimize the emissions from the ISI. More so, agricultural applications of biochar include improve soil nutrient retention, soil structure, and water-holding capacity, enhancing crop yields and facilitating climate change mitigation. The conversion of agricultural wastes into biochar alleviates the environmental challenges resulting from the dumping and burning of agricultural and forest wastes in an open field (Ibitoye et al. 2021a, b). This creates valuable products from waste, promoting a circular economy. According to research, biochar can potentially restore contaminated land for agricultural use by lowering the bioavailability of pollutants and heavy metals in soil (O’Connor et al. 2018). It is a dependable method of cleaning up a variety of contaminants from contaminated soil due to its large surface area, surface functional groups, and sorption capacity (Issaka et al. 2022). The use of biochar in soil remediation is further supported by its capacity to absorb organic contaminants and heavy metals, as well as by its capacity to raise soil pH and stabilize heavy metal concentrations. Specifically, it has been discovered that applying biochar in situ can effectively lower the mobility of heavy metals in soils (Singh and Singh 2020).

The adoption of sustainable practices, such application of biochar, may give the ISI a competitive edge (Gąsior and Tic 2017). This can be adopted as a marketing and sensitization tool, especially where sustainability is a top priority for stakeholders. Venture capitalists are attracted to industries that are committed to sustainability, especially investors that prioritize environmental, social, and governance. Investing in biochar technology might be interpreted as a sign of responsible and progressive management (Hyytiä 2022).

The utilization of biochar in iron and steel production can improve air quality and the well-being of the people living around the industrial communities (Wang et al. 2023b). This is vital in industrial areas where air pollution is a serious problem. The production and application of biochar encourage the creation of employment. The biomass collection, biochar production, and distribution generate new employment, thus improving the standard of the people, and minimizing social vices resulting from the unemployed populace (Cha et al. 2016). By utilizing agronomic and economic models, Dumortier et al. (2020) assess farmers’ 20-year willingness to invest in biochar. The benefits include increased revenue and potential policy gains, especially if biochar production is paired with biofuel production or efforts to reduce greenhouse gas emissions. Collaboration and open communication with local communities and investors are essential to the long-term sustainability of biochar production for industrial applications.

## Regulatory and environmental considerations

Integrating biochar production and application in the ISI is not just a technologically challenged endeavor but also involves sailing through the relevant regulations and dealing with sustainability challenges (Campos and Assis 2021; Chang et al. 2023). Environmental and regulatory difficulties are critical to evaluating the practical application of biochar and prolonging the viability of the business. Detailed regulatory frameworks for integrating biochar in ISI are listed as follows:

Quality standards for ISI: Using biochar in the ISI must not lower the quality of the output products. For instance, the mechanical, chemical, and surface properties of the steel produced must be thoroughly examined to meet industry standards. Compliance with international standards highlights the importance of assuring the reliability and quality of steel products.

Resource efficiency and material compatibility: It is important to assess the efficiency of utilizing biochar in iron and steel production, considering the energy utilization and purchase of feedstock. It is essential to ensure that biochar is compatible with existing raw materials and production processes to advance general efficiency and incorporation into the iron and steel industry.

Workplace health and safety: adhering to workplace health and safety rules and regulations when utilizing biochar in iron and steel production processes is essential. This includes applying measures to safeguard staff, recognizing likely dangers related to biochar use, and making sure staff adheres to approved health and safety rules within the ISI (Azzi et al. 2022; Mehmood et al. 2023; Pourhashem et al. 2019).

Emission reduction strategies: The CCS potential of biochar can be leveraged in the industry’s emission reduction plans (Burezq and Davidson 2023). The obligation of the ISI to reduce CO_2_ emissions supports the worldwide climate goals (IPCC 2022; REN21, 2022). Integrating biochar into emission reduction plans can lead to measurable benefits, including minimizing dependence on fossil-based fuels.

Life cycle assessment (LCA): A comprehensive LCA evaluates the impact of biochar generation and use in the ISI from the cradle to the grave (Azzi et al. 2022; Nurdiawati et al. 2023). This evaluation considers resources, energy use, emissions, and potential environmental benefits. The LCA results provide valuable insights into the overall environmental impact of biochar integration in the ISI.

## Recent research on the use of biomass/biochar in ISI

The recent research efforts on biomass and biochar production and use in ISI are presented in the section. Biomass and biochar utilization are discussed, including reducing agents, emission reduction, co-firing, and operating cost reduction potentials.

### Substitution/co-combustion

Several studies have been carried out on developing substitutes for fossil-based fuels. The use of biochar as a replacement for coal and coke has been studied with a particular focus on the metallurgical, technical, and environmental requirements (Safarian 2023b). Research has revealed that biochars produced from wood and wood residues can potentially substitute coal and coke in iron and steel making, which is connected to their favorable physicochemical properties (El-tawil et al. 2021). It is feasible to create bio-coke by mixing biochar with coal during coke-making. However, the amount of biochar must be kept between 2 and 10% to prevent adverse effects on the quality of the final coke (bio-coke) (Safarian 2023b). These values strongly depend on the biochar particle size and FC concentration. Moreover, when biochar particle size is reduced, both the CSR and the CRI decrease, yet there is a slight improvement in fluidity. As a result, the most effective particle size range for biochar was identified to be between 2 and 4 mm (Safarian 2023b). It was further opined that other biochar types may require evaluation, especially the FC content and heating value properties, to determine their potential to substitute coal and coke.

In the sintering process, biochar presents significant potential for replacing coke breeze (a smaller particle-sized coke). Using coke breeze in sintering accounts for 9–12% of the total energy consumption and 12% of the greenhouse gas emissions in steel-making plants (Ye et al. 2019). Safarian’s (2023b) study highlighted that substituting 40–60% of coke breeze with biochar maintains a high-quality sinter and a product yield exceeding 80%. Adding up to 60% biochar into the sintering plant achieved a product yield comparable to that obtained with 100% coke breeze. Gan et al. (2012) and Lu et al. (2013) demonstrated that partially substituting coke breeze with biochar as an alternative fuel in a sintering process leads to increased CO and CO_2_ values while reducing SOx and NOx emissions.

A technology blending coke and biochar was recommended to augment fuel distribution during iron ore sintering (Wang et al. 2023a). The iron ore sintering process was enhanced by optimizing fuel distribution and adjusting the biochar ratio in the upper and lower bed layers to improve thermal distribution. This involved reducing biochar replacement in the upper layer and increasing it in the lower layer. The study also investigated quasi-particle granulating characteristics under varying biochar replacement ratios, while experimental monitoring tracked temperature changes in upper and lower layer beds during the sintering process. According to the results, the melting temperature and melting amount index of the upper and lower layers following the addition of divided fuel are significantly greater than those without breakdown. During the sintering, the highest substitution ratio of biochar increased by 25%, significantly lowering the amount of coke used and the amount of carbon emissions (Wang et al. 2023a).

El-tawil et al. (2021) examined coking coal blends with 5 and 10% additions of biochar created at different temperatures and different origins to investigate the effect of biochar on the characteristics and reactivity of the cooking coal blend. Also, investigations were done to comprehend the effect of biochar addition on plasticity. Types of bio-coke produced at a technical scale (closely resemble those found in an actual industrial or operational setting within the steel production process) demonstrated promising results in standard tests evaluating reactivity, strength after reaction (ability to withstand chemical reactions without significant deterioration in its physical properties), and mechanical strength (ability to resist deformation, breakage under applied forces or loads). These findings suggest that a coking coal blend incorporating 5% high-temperature torrefied biochar could prove suitable for industrial applications within coke-making processes (El-tawil et al. 2021). Moreover, there was no noteworthy variation between the qualities of coking coal blends with 5% biochar addition generated on a technical scale and in a laboratory regarding reactivity as determined by TGA.

The utilization of biochar as a replacement for fossil coal in EAF has been reported in the literature, where biochar is produced in different processes like torrefaction, slow pyrolysis, and HTC (Cardarelli et al. 2022; Ye et al. 2019). In EAF steel-making, scrap is melted using electric energy and supplemented by natural gas and coal. Natural gas powers specialized burners for scrap melting, while coal, mainly anthracite, serves multiple roles-it acts as a charged carbon in the basket, consuming excess oxygen and providing heat during melting (Cardarelli et al. 2022). Pulverized coal, injected via wall injectors, interacts with oxygen to create protective slag, reducing electricity usage and safeguarding equipment. Additionally, coal acts as an alloying element in molten iron for steel production. The use of biochars as a substitute for coal in the EAF steel manufacturing process did not result in appreciable detrimental changes, according to the results (Cardarelli et al. 2022). Faster heat release from highly reactive biochars encourages the rise of temperatures within the EAF, which lowers the electrical demand and energy utilization from the electrodes. Using biochar as a reducing agent and foaming slag enhancer may be appropriate, especially biochar that produces reducing gases at higher temperatures (Cardarelli et al. 2022; Kusch-Brandt 2018). It was also claimed that biochar with a more significant proportion of FC and a smaller proportion of AC and VM would be more likely to be utilized as an iron carburizer (Cardarelli et al. 2022).

Robinson et al. (2021) lay the groundwork, experimentally and practically, for incorporating renewable biochar into the EAF to mitigate the climate impact of steel production. Lab-scale tests were conducted using four different carbonaceous materials, including synthetic graphite, anthracite coal, and 2 different biochar-generated from wood, to assess biochar’s performance as a carburizing agent. The dissolution rate constants observed in these experiments ranged from 1 to 2 × 10 –4 m/s, aligning well with previously reported findings. Additionally, the lab-scale results indicate that properties often considered unfavorable in biochar, such as high porosity and low carbon crystallinity, may not hinder its effectiveness as a carburizer in steel-making. A fifty-ton electric arc furnace was used for an industrial trial that involved six successive heats. The results revealed that replacing 33% of the standard anthracite carbon charge with biochar did not affect the electric arc furnace’s normal working conditions (Robinson et al. 2021).

The characteristics of biochar produced from biomass–coal hybrid fuel with 30% biomass (weight/weight) were studied using experimental methods. Compared to the 27 MJ/kg of coke breeze, the heating values of the developed biomass–coal hybrid fuel was about 28 MJ/kg (Reis et al. 2023). Proximate analysis indicated that the biochar samples exhibited higher VM contents compared to coke breeze, although the VM contents of the biochar are lower than those of anthracite coal. The results indicated that utilizing biomass–coal hybrid fuels could replace some of the coke breezes in the sintering and EAF processes. This substitution can potentially play a substantial role in reaching net-zero objectives of up to 30% CO_2_ emissions reduction.

### Application of biochar as a reducing agent

Reducing agents manufactured from biomass materials is viewed as one potential option in pursuing strategies to reduce fossil CO_2_ emissions. Utilizing a biomass-based reducing agent can significantly reduce the life cycle emissions of the steel-making process (Suopajärvi et al. 2017). A review on using biochar as an energy carrier or a reducing agent in Europe and America showed that biochar produced from various biomass types can serve as a reducing agent (Kusch-Brandt 2018).

Mousa et al. (2017) looked at biomass lignin-bonded briquettes as a reducing agent in a BF. The study used lignin as an alternative to cement to make both wholly and partially briquettes. Up to 25% of the cement was replaced with lignin, resulting in sufficient-strength briquettes for BFs.

Biochar as an additional reductant in the BF was investigated using a numerical approach (Wiklund et al. 2016). The necessary pre-processing of biomass for biochar generation were also examined, emphasizing energy consumption and process economics. Utilizing heat from hot stove flue gases and burning BF top gas as primary heat sources for biochar production were compared as two preheating concepts. The findings indicated that, for a facility with steel production of about 1 Mt per year, using hot stove flue gases for biochar production lowers the yearly working expenses of the preheating biomass by around 0.5 M€ (Wiklund et al. 2016).

### Operational cost and CO_2_ reduction potential

The use of biochar as a CO_2_ reduction technique in the ISI has been a research focus (Hanrot et al. 2009). According to the research of Feliciano-Bruzual and Mathews (2013), the injection of powdered biochar particles into the tuyeres of BFs represents an appealing and logical approach to reducing the CO_2_ emissions produced while manufacturing substantial hot metal.

Norgate et al. (2012) examined the suitability of biomass as a renewable source of biocarbon for iron and steel production. The findings showed that using biochar in the incorporated steel-making pathway lowers the carbon footprint of steel by 31–57% without any credits for the byproducts of charcoal production and by 42–74% with these credits included. However, the amount of the byproduct contribution relies on the retort byproduct yields of the biochar, which in turn depend on various variables, including the characteristics of the thermochemical treatment process and the composition of the feedstock (Norgate et al. 2012).

Zang et al. (2023) explored decarbonization solutions for BF-BOF and EAF processes to decarbonize the ISI. The potential for CO_2_ reduction for each decarbonization strategy using life cycle analysis and the related costs using techno-economic analysis were investigated. According to the study, BF-BOF and EAF cradle-to-gate CO_2_ emissions can be decreased to 16 kg/MT steel and 25 kg/MT steel when combined with biomass-based energy sources. Depending on the different approaches to decarbonization and energy prices, the projected CO_2_ prevention costs (economic costs involved in implementing technologies or measures to prevent or reduce CO_2_ emissions) range from $90 to $646/MT CO_2_. Similarly, a thorough analysis of the current iron and steel output and assessing the decarbonization methods were carried out (Fan and Friedmann 2021). The DRI and EAF appears to have a superior decarbonization ability to go toward net-zero emission. In contrast, BFs coupled with basic oxygen furnaces exhibit limited compatibility with decarbonization technology.

Wang et al. (2020) pioneered the idea of mass-thermal network optimization in the ISI. The report also compiles reports on cases and initiatives for demonstration from throughout the globe. It was established that the best energy target for the ISI should be made by applying several production methods, including efficient and sustainable technology, such as biochar. It was further opined that selecting predetermined extreme operating parameter values will result in optimal energy savings.

Biomass combustion focuses on experimental and numerical investigations and how they might be used to optimize BFs was conducted by Liu and Shen (2021). According to the study, pulverized biomass injection is a reliable way to achieve consistent, high productivity, low cost, and low CO_2_ iron-making in BFs. Another research revealed that using residual biomass, such as agricultural waste, might drastically lower production costs for biochar production by 120–180 USD/t compared to generating biochar using woody biomass (Feliciano-Bruzual and Mathews 2013).

A theoretical study compared natural gas use in an EAF steel-making process with rice husk, coffee husk, and elephant grass (Luís and Santos 2015). Three scenarios were suggested, each varying equipment efficiencies (varying operating pressure and temperature). The Rankine cycle was employed in three different situations utilizing biomass and natural gas. The energy comparison investigation revealed that natural gas fuel use is the lowest among the 3 cases and demonstrates minimal variations relative to them. According to an economic analysis that only considered the plant’s operating costs, elephant grass had the lowest cost of operation. This occurs because the utilization of biomass results in a larger volume of exhaust gas compared to natural gas, primarily due to the lower heating value of the fuels. Elephant grass, for instance, has a lower calorific value of 17 MJ/kg, notably smaller than that of natural gas at 47 MJ/kg. Despite the lower heating values of biomasses compared to natural gas, they showed promise for use in the EAF, suggesting their viability as an excellent substitute for natural gas in EAF iron making.

A detailed review has been done on various alternative fuels to address ecological and energy-efficient concerns: biomass, hydrogen fuels, and recovered carbon reserves for coke breeze in iron ore sintering (Cheng et al. 2020b). Analysis of fuel reactivity and complementing characteristics of the flame front speed and heat front speed was done to determine the detrimental effects of substitute fuel on the heat trend and sinter effectiveness, mainly when there is a high substitution rate. It was suggested that assessments of the essential properties of other types of renewable unconventional fuels with substantial sources and significant FC be made. Also, investigations of the effects of alternative fuels on the efficiency and emissions of sintering should be carried out. It is also vital to do economic studies of renewable fuel alternatives (Cheng et al. 2020b).

An economic study has considered the practical applications of BF-BOF, especially where charcoal injection takes the role of pulverized coal. In the integrated BF-BOF route, incorporating renewable biochars in iron and steel production can reduce net CO_2_ emissions by 32–58%, with even greater benefits under full life-cycle considerations. However, the possible reduction in the EAF route is lower, approximately 10–15%, as its energy primarily relies on the electricity grid and is contingent on emissions within that sector (Jahanshahi et al. 2014). According to this study, the net cost of manufacturing biochar, the choice of pyrolysis method, the value of byproducts, and the value of the biochar itself are the main economic determinants (Jahanshahi et al. 2014).

### Recent technological innovations in biochar production and utilization

Recent biochar production and utilization advancements have focused on improving reactor design, optimizing processes, and tailoring biochar properties to meet specific industrial requirements, such as those of the steel industry. Numerous advanced biochar production techniques, such as vacuum pyrolysis, HTC, microwave pyrolysis, electro-modified methods, and magnetic biochar production, have gained recognition for their efficiency in heating biomass uniformly, leading to higher yields and improved quality (Adeniyi et al. 2023; Bhatt et al. 2022; Danesh et al. 2023; Dermawan et al. 2022; Ying et al. 2023). The recent research on these advanced biochar production methods has been reported in the literature; however, the brief description and their key advantage are enumerated in Table 5. Vacuum pyrolysis involves low-pressure thermal degradation, yielding high-quality biochar with enhanced porosity. HTC converts high-moisture feedstocks into biochar without pre-drying, preserving nutrients and reducing the oxygen-to-carbon ratio. Microwave pyrolysis offers rapid, uniform heating, reducing temperature requirements. Electro-modification enhances biochar’s adsorption properties, while magnetic biochar exhibits high adsorption capacity and easy recovery. These techniques find diverse applications in agriculture, waste management, and environmental remediation, offering potential benefits for steelmaking where consistent biochar properties are crucial.

**Table 5:** Recent technological innovations in biochar production (Adeniyi et al. 2023; Bhatt et al. 2022; Danesh et al. 2023; Dermawan et al. 2022; Horst et al. 2023; Ying et al. 2023; Zubair et al. 2022)

Production Technique	Description	Key Advantages
Vacuum pyrolysis	Thermal degradation of biomass under vacuum or low pressure.	- High-quality biochar production - Effective vapor removal - High porosity
Hydrothermal carbonization	Convert high-moisture feedstocks into biochar at high temperatures and pressures.	- Retained nutrients - Reduced oxygen-to-carbon ratio - Enhanced properties
Microwave pyrolysis	Rapid heating of biomass using microwaves results in biochar with improved properties.	- Reduced temperature requirement - Rapid and uniform heating - Improved gasification
Electro-modified biochar	Chemical treatment of biochar with an electric field to enhance adsorption properties.	- Increased surface area - Enhanced specific adsorption - Improved functionality
Magnetic biochar	Preparation of biochar with magnetic properties for efficient pollutant removal and recovery.	- High adsorption capacity - Easy separation and recovery - Versatile applications
Plasma pyrolysis	Subjecting materials to extremely high temperatures using a plasma torch or arc discharge.	- Effective for processing complex materials - Produces carbon-rich biochar - Used in hazardous waste remediation, -Used in materials synthesis and energy production.

#### Reactor design

Traditional batch reactors have limitations in terms of scalability and efficiency (Beston 2024; Doing 2024). Reactor design parameters such as temperature and residence time significantly influence the physico-chemical properties of biochar (Adeniyi et al. 2023; Moser et al. 2023). Higher temperatures and longer residence times typically result in biochar with higher carbon content and lower VM, which are desirable properties for steelmaking applications (Ibitoye et al. 2021b; Wang et al. 2022). Research has shown that the reactors equipped with catalysts can modify the pyrolysis process, leading to biochar with specific properties tailored for different applications. Catalysts can enhance biochar yield, improve its chemical composition, morphological properties, and reduce impurities such as ash and tar, thereby enhancing its suitability as a reducing agent or additive in steelmaking processes (Cao 2017; Wang et al. 2022).

Biochar produced in specialized reactors can be an ISI renewable and carbon-neutral reducing agent. By substituting fossil fuels like coke or coal with biochar, steelmakers can reduce their carbon footprint and reliance on finite resources while maintaining or improving process efficiency.

Continuous pyrolysis reactors have recently been developed, offering advantages such as higher throughput, better temperature control, and improved energy efficiency (Ünsaç et al. 2024; Xu et al. 2021). These reactors allow for a continuous feed of biomass, resulting in a steady output of biochar (Beston 2024). Batch reactors, on the other hand, are suitable for smaller-scale operations but may lack the throughput required for industrial applications (Qureshi et al. 2018). The continuous pyrolysis plant outperforms batch and semi-continuous models with its larger capacity, enhanced automation, and eco-friendliness (Beston 2024; Qureshi et al. 2018; Ünsaç et al. 2024; Xu et al. 2021). It operates smoothly with minimal manual involvement, employs hot air heating for efficiency, and incorporates advanced de-dusting and condensation systems. It guarantees uninterrupted operation, making it well-suited for large-scale waste processing (Beston 2024).

**Fig. 14:**
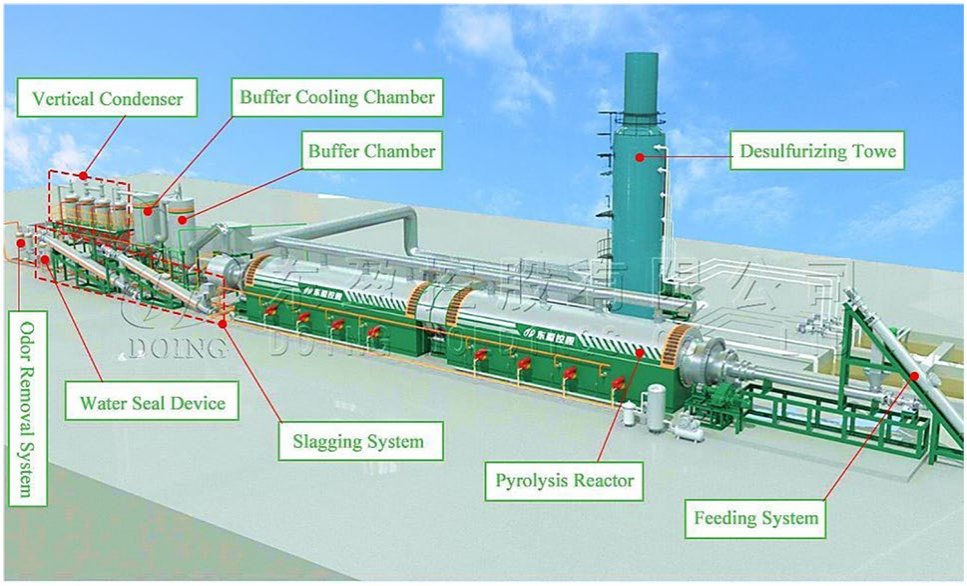
A typical continuous pyrolysis plant. Reprinted from Doing (2024) (Copyright © 2024, with permission from Henan Doing Environmental Protection Technology Co., Ltd.)

Doing (2024) has introduced the continuous waste plastic pyrolysis plant. This cutting-edge system (Fig. 14) enables the uninterrupted conversion of plastic into valuable products such as plastic pyrolysis oil and biochar. Utilizing a Programmable Logic Controller (PLC) simplifies all operations of the continuous pyrolysis plant, offering significant time and labor savings. The critical components of the plants include the pyrolysis reactor, condenser, and PLC for efficient management.

#### Biochar production process optimization

Process optimization techniques, encompassing adjustments in temperature profiles, residence times, and feedstock characteristics, are pivotal in maximizing biochar yield. Augmented biochar yield per unit of feedstock enhances production efficiency and reduces overall production costs, making it economically feasible for expansive applications like steelmaking (Ochieng and Cer 2023; Qureshi et al. 2018). Precise control over process parameters enables biochar production with tailored properties conducive to steelmaking, notably high carbon and low ash content. Pyrolysis optimization minimizes impurities and enhances biochar’s physical and chemical traits, heightening its efficacy as an additive or reducing agent in steel production (Christian et al. 2021; Gupta et al. 2023; Hossain et al. 2017).

Strategies for process optimization include adopting energy-efficient heating methods like microwave-assisted pyrolysis or integrating biochar production with other processes to leverage waste heat and reduce energy consumption and greenhouse gas emissions related to biochar production. This convergence resonates with the sustainability objectives of the steel industry, amplifying biochar’s environmental credentials as a feedstock or additive.

Tailoring the pyrolysis conditions allows for biochar production with targeted particle size distribution, surface areas, and reactivity profiles, optimizing its utility as a reducing agent in blast furnaces or as an additive in iron ore pelletization. By optimizing process parameters to maximize biochar yield and quality while minimizing energy consumption and production costs, biochar emerges as a more economically competitive alternative to conventional carbon sources in steelmaking. This enhances its attraction as a sustainable feedstock or additive in the steel industry, propelling broader adoption (Christian et al. 2021).

#### Tailored biochar properties for the steel industry

Advanced biochar production techniques offer tailored properties crucial for optimizing biochar’s effectiveness in steel industrial utilization. By meticulously adjusting parameters such as temperature, heating rate, and feedstock composition, these methods can significantly enhance biochar’s carbon content, a critical factor influencing its reactivity and substitutability for traditional carbon sources like coke or coal in steelmaking processes (Chen et al. 2023; Mohit and Remya 2023). Specifically, higher carbon content biochar exhibits increased reactivity, making it an efficient substitute for coke or coal in reducing iron oxides during steel production. Moreover, precise control over process parameters ensures the production of clean biochar with minimal ash, volatile organic compounds, and heavy metals. This low AC is vital for maintaining the purity of steel products and preventing contamination during the steelmaking process, ultimately contributing to the production of high-quality steel products.

Tailoring particle size distribution is another essential aspect facilitated by advanced biochar production techniques. By optimizing grinding, sieving, or granulation processes, biochar producers can achieve a particle size distribution that matches the specific requirements of steel-making processes, such as blast furnace injection or pelletization (Ibitoye et al. 2023a, b; Khanna et al. 2019). This ensures uniform distribution and efficient utilization of biochar, maximizing its effectiveness as a reducing agent or carbon source in steel production. Furthermore, advanced biochar production techniques enable the modification of biochar surface chemistry to enhance its interaction with metals and other materials in the steel-making process (Amalina et al. 2023). Surface functionalization or activation methods introduce specific chemical groups or catalysts onto the biochar surface, improving its adsorption capacity, reactivity, and catalytic properties. This tailored surface chemistry optimization can significantly enhance biochar’s performance as an additive, catalyst, or adsorbent in steel-making processes, leading to improved efficiency and product quality (Amalina et al. 2023; Cao 2017; Conte et al. 2021).

Enhanced thermal stability is also a crucial aspect facilitated by tailored biochar properties. Biochar producers can increase biochar thermal stability by optimizing carbonization and activation processes, making it more resistant to high temperatures and harsh conditions in steel-making processes (Amalina et al. 2023; Cirilli et al. 2018). This ensures consistent performance and prolonged lifespan of biochar in steel-making applications, contributing to the overall efficiency and reliability of the steel production process.

Tailored biochar properties enable the development of customized additives designed explicitly for steel-making applications. Advanced biochar production techniques allow for incorporating additives such as minerals, metals, or functional groups into the biochar matrix, imparting desired properties for various steel-making processes. These customized additives can enhance biochar’s performance as a reducing agent, flux, binder, or catalyst in steel-making processes, ultimately improving process efficiency, product quality, and environmental sustainability (Conte et al. 2021; Giorcelli et al. 2019; Tu et al. 2017).

#### Some innovative biochar utilization

The innovative functionalization strategies highlight the potential of biochar as a versatile material for addressing various environmental and agricultural challenges. Tailoring biochar properties through functionalization can effectively be utilized in diverse applications. These include remediation, composite materials, and soil improvement, contributing to sustainability and resource efficiency. Other innovative biochar use is as follows:

Magnetic biochars: Magnetic biochars are created by incorporating magnetic nanoparticles, such as Fe_2_O_3_ and Fe_3_O_4_, onto the surface of biochar (Conte et al. 2021; Tu et al. 2017). This functionalization allows easy biochar removal from soils and water using a magnetic field. Two main pathways are used: treating biomass with iron-containing solutions before pyrolysis/HTC or synthesizing magnetic nanoparticles directly on the biochar surface (Conte et al. 2021; Frolova 2019). Magnetic biochars facilitate removal and exhibit improved physico-chemical properties, such as increased surface area and porosity, leading to enhanced adsorption capacity for contaminants.

Plasticized biochars: Plasticization involves combining biochar with epoxy resin and hardener to produce composite materials with improved properties (Conte et al. 2021; Giorcelli et al. 2019). These biochar-based plastics exhibit increased elasticity or ductility depending on the amount of biochar added. Adjusting the biochar content can produce a wide range of products with tailored mechanical properties. Giorcelli et al. (2019) combined biochar with a low-viscosity epoxy resin and a hardener to create a composite material. This composite exhibited increased elasticity when the biochar content was below 2% (w/w). This suggests that biochar-based plastics could manufacture various products depending on the specific properties required, such as elasticity or ductility.

Co-composted biochar: The emergence of multiple nutrient deficiencies resulting from soil fertility depletion in various regions across the planet poses a significant challenge to the sustainability of agriculture on a global scale. As a result, biochar has been recognized as a beneficial amendment for enhancing soil quality (Amalina et al. 2023; Danesh et al. 2023). It can impact soil structure, improving water retention and nutrient availability (Conte et al. 2021). Co-composting involves mixing organic wastes with biochar to produce compost with enhanced nutrient retention and release properties. Co-composted biochar exhibits a high capacity for capturing and releasing nitrates and enhanced cation exchange capacity (Conte et al. 2021). Additionally, the co-composting process reduces potentially harmful chemicals and enhances soil fertility. Incorporating biochar into compost becomes a valuable amendment for improving soil quality and promoting crop production (Amalina et al. 2023; Danesh et al. 2023).

## Biochar integration strategy in the steel industry

While specific case studies on biochar integration in the steel industry might be limited, using biochar as a reducing agent, carbon sequestration tool, etc., aligns with industry trends. Successful implementation would likely involve the following steps:

i. Pilot studies: Industry stakeholders could conduct pilot studies to assess the feasibility of integrating biochar production and utilization in steel-making processes. These studies would evaluate factors such as biochar’s impact on reduction reactions, emissions, and product quality (Hammerschmid 2021).
ii. Customization: Characteristics of biochar can be adapted to specific steel-making processes. To enable optimal integration, the reactivity, combustion properties, and injection methods of biochar would need to be optimized (El-tawil et al. 2021; Feliciano-Bruzual and Mathews 2013; Feliciano and John 2014).
iii. Process integration: Incorporating biochar would necessitate changes to current steel production techniques. Steel industries and biochar producers must work together to develop efficient and successful integration techniques.
iv. Economic analysis: Conducting detailed economic feasibility assessments would aid in determining the costs and advantages of biochar integration. Evaluating potential cost savings, income streams, and environmentally friendly offset opportunities will inform decision-making (Salimbeni et al. 2023; Wiklund et al. 2016; Yang et al. 2023).
v. Regulatory compliance: Ensuring no violations of environmental laws and emission requirements would be vital. Engaging with regulatory agencies will promote approvals and permit purchases (Pourhashem et al. 2019).
vi. Stakeholder engagement: Collaboration with stakeholders, including regulators, shareholders, and communities, would help to increase support for biochar integration activities.
vii. Knowledge sharing: Successful implementation would involve sharing knowledge and best practices across the industry to encourage broader adoption.

## Prospects and research directions

Using biochar in steel-making operations holds enormous potential for alleviating the industry’s environmental concerns. The steel industry may drastically reduce its carbon footprint by using biochar as a reducing agent or alternative carbon source in BFs. Notwithstanding its significance in cutting emissions, biochar’s sustainable source from biomass corresponds with the worldwide transition toward green and circular economy practices (Danesh et al. 2023; Foong et al. 2023). Furthermore, replacing or supplementing coke with biochar in the BF addresses environmental problems. It helps the sector remain resilient despite resource constraints and shifting commodity costs.

### Development of innovative manufacturing techniques

Traditionally biochars are used for carbon sequestration and soil amendment. Dumortier et al. (2020) reported significant yield increases by using biochar for soil remediation. Biochar also found applications in several cutting-edge and future industries.

#### Supercapacitors and batteries

The utilization of biochar in the development of high-performance supercapacitors has demonstrated potential owing to its substantial surface area and advanced micro-mesoporosity (Xiu et al. 2019). It may be made from different biomass sources, including chicken dung, and activated to form a highly specific surface area hierarchically porous structure. This makes it a great electrode material for supercapacitors (Pontiroli et al. 2019). To create biochar with a high specific surface area from marine biomass waste, a simple and environmentally friendly one-step procedure has also been developed, increasing the material’s potential for energy storage applications (Gao et al. 2021). All of these researches demonstrate how biochar may be used to create supercapacitors and is a viable and affordable substitute for conventional materials.

#### Water and air purification

Biochars have high adsorption capacity, which makes them good materials for water and air purification in domestic and industrial settings. Biochar made from rice husk, wheat straw, and corncob has shown strong adsorption capabilities for lead and cadmium, demonstrating the material’s efficacy in removing heavy metals from wastewater (Amen et al. 2020). It has been discovered that engineered biochar, which has been altered to improve its qualities, is very effective at eliminating impurities from water (Akhil et al. 2021). It has been demonstrated that biochar can be used as an inexpensive bio-adsorbent to remove pollutants, such as organic compounds and heavy metals; however, the effectiveness of this process depends on the manufacturing processes and feedstock used (Fdez-Sanromán et al., 2020).

#### Concrete and asphalt additives

The capacity of biochar to improve mechanical properties like strength and durability is making the application of biochar as construction materials increasingly popular, especially in concrete and asphalt productions (Kamini et al. 2023; Singhal 2023). According to Singhal (2023), adding biochar to concrete at a rate of 1–3% from different lignocellulosic biomass can serve as an economical and environmentally beneficial alternative to binders. In addition, Aman et al. (2022) highlight how biochar can be used to replace cement in concrete composites, increasing their strength and other characteristics. According to Gupta and Kua (2017), biochar has been shown to have environmental advantages, including the capacity to sequester carbon. These studies highlight how biochar has a lot of potential for use in the building sector, especially when it comes to improving the sustainability and performance of construction materials.

To effectively integrate biochar into the ISIs, several key areas required further research and development efforts. The scarcity of recent and publicly available data on the economic realities of biochar use in iron-making processes poses a challenge in accurately assessing the opportunities for biochar integration. Thus, undertaking comprehensive economic studies on biochar application in the ISI is imperative. These studies would provide recent insights into the viability and economic feasibility of incorporating biochar into existing industrial processes.

Addressing commercial difficulties connected to feedstock optimization, scaling up production, and conducting comprehensive sustainability assessments across the biochar life cycle is essential. This entails exploring advanced biochar production methods tailored explicitly for iron and steel industrial applications. These methods should prioritize optimizing efficiency, cost-effectiveness, and the quality of biochar produced. Furthermore, increasing the implementation and acceptability of biochar within ISIs necessitates addressing concerns related to performance, economic viability, and regulatory compliance. Conducting thorough life cycle analyses and technical and economic evaluations can help to demonstrate further the economic and environmental benefits of integrating biochar into iron and steel processes. Additionally, engaging with stakeholders in the ISI through knowledge-sharing platforms, collaborative investigations, and industrial demonstrations is crucial. This collaborative approach can foster confidence in biochar technology and facilitate its widespread adoption and implementation within the ISI.

Biochar’s potential to serve as a carbon sink and mitigate climate-related challenges by sequestering CO_2_ from the atmosphere holds significant promise. However, its efficacy hinges on various factors, including feedstock compositions and production methods. Understanding these nuances requires further research to establish clear correlations between biochar properties and soil-crop responses in different environmental conditions.

### Some speculative biochar applications

The development of advanced nanocomposites with improved properties may be possible using biochar as additive materials. A recent study supports the potential of biochar as an additional material for the production of sophisticated nanocomposites with enhanced mechanical and electrical characteristics. Veličković et al. (2019) and Kausar (2020) emphasize the advantages of nanocomposites in the electronics and automotive sectors, respectively, and how biochar may improve these characteristics. The potential of biochar-supported nanomaterials in environmental applications is further highlighted by Rodriguez-Narvaez et al. (2019), who propose various possible applications for nanocomposites based on biochar. Chausali et al. (2021) provide more evidence for the ability of biochar to improve the properties of nanocomposites by addressing in detail the applications of nanobiochar and biochar-based nanocomposites in agriculture and the environment.

Previous studies have highlighted the potential of biochar for sustainable and biodegradable food packaging materials that can help increase the shelf life of food  products (Al-Tayyar et al. 2020; Asgher et al. 2020; Nilsen-Nygaard et al. 2021; Wang et al. 2021). Its capacity to absorb moisture is well recognized, and it can help minimize food waste by preventing food from spoiling. Furthermore, research on biochar’s effectiveness as a protection against foodborne pathogens has increased (Al-Tayyar et al. 2020; Asgher et al. 2020). This finding adds to the material’s potential applications including food packaging. However, more investigation is required to completely comprehend the working principles and enhance biochar’s effectiveness as a material for food packaging (Wang et al. 2021).

According to research, textiles can be treated with biochar to offer antibacterial, deodorizing, and UV protection properties (Reta et al. 2024). Sportswear, medical fabrics, and outdoor gear are possible markets for this. In addition to improving drying qualities, odor adsorption, moisture transfer, and air and vapor permeability, biochar can also be used in textiles (Çay et al. 2020).

## Conclusion

Investigating the use of biochar in the iron and steel industry reveals a possible path toward sustainability and innovation. This research illustrates the production and extensive potential of biochar across numerous steel industrial uses, such as soil enhancement, waste transformation, storage of carbon, and reducing agents. The characteristics of biochar were compared with coal and coke. Slow pyrolysis and hydrothermal carbonization are the most effective techniques for producing high-yielding biochar, with yields that vary from 25 to 90%. Biochar has 1–5% moisture content, comparable to coke (1–10%) but significantly lower than coal (10–15%). Also, it has a 10–12% volatile content, comparable to coke (1–2%) but lower than coal (15–30%). Biochar has a high fixed carbon percentage, ranging from 85 to 87%, comparable to coke (85–88%) and substantially higher than coal (50–55%). It has a low ash content of roughly 3%, equivalent to coal (ash content of 1%) and substantially lower than coke (12.9%). Biochar has a lower bulk density than coal and coke, ranging between 180 and 240 kg/m^3^, whereas coal and coke have bulk densities of 800–850 kg/m^3^ and 400 to 500 kg/m^3^, respectively. Moreso, the biochar heating value ranges from 30 to 32 MJ/kg, comparable to coke (30 MJ/kg) and exceeding coal (23 MJ/kg). Biochar has a high porosity of around 58.22%, which surpasses the porosity values observed in coal (10%) and coke (2%).

Significant reductions in CO_2_ emissions (32–58%) are attainable, primarily by BF tuyere injections. When BF nut coke is replaced with biochar with less than 7% volatile content, high density, and particle sizes ranging from 20 to 25 mm, CO_2_ emissions are anticipated to be 3–7% lower. In iron ore sintering, biochar, particularly with low volatile, high densities surpassing 700 kg/m^3^, and small particles ranging from 0.3 to 3 mm, can result in CO_2_ reductions of 5–15%. Furthermore, when utilized as a BF injectant, biochars with volatile between 10 and 20%, ash content less than 5%, and low alkali levels can generate significant CO_2_ emissions savings of 19–25%. While biochar holds versatile promise, its successful integration into the steel sector necessitates alignment with existing processes, technical advancements, and operational efficiency. Regulatory adherence, environmental assessments, and emissions control are imperative for sustainable and compliant integration.

Biochar is vital in several applications, such as water treatment and soil fertility improvement. It enhances composting by improving water retention aeration and facilitating microbial decomposition. Its porous structure effectively removes heavy metals and organic pollutants from water and soil, aiding environmental cleanup. When added to soil, biochar improves water retention and soil fertility and helps remove contaminants, benefiting crop growth and land restoration. As a high-value fuel source, biochar contributes to renewable energy initiatives. In agriculture, nano-biochar shows potential for slowly releasing fertilizers and optimizing nutrient management practices.

Integration of biochar will impact the steel and allied industries. Steel producers can transition to sustainable reducing agents and carbon emissions sequestration, aligning with United Nations sustainable development goals (SDGs), particularly SDGs 7 (affordable and clean energy), 13 (climate change), and circular economy principles. The effective use of biochar could serve as a model for other sectors seeking advanced, eco-friendly solutions.

Beyond its immediate utility, biochar holds vast potential as a hallmark of sustainable development, circular economy, and resource efficiency.

Amidst environmental challenges and resource constraints, biochar emerges as a beacon of hope and opportunity in the steel industries. Realizing the full potential of biochar demands collaborative teamwork, research, development, and unwavering commitment to shaping a more sustainable future.

### Electronic supplementary material

Below is the link to the electronic supplementary material.

Supplementary Material 1

## Data Availability

All data used for the study are presented within the manuscript.
