# Supplementary figures and images for: An overview of biochar production techniques and application in iron and steel industries
Source: Bioresour Bioprocess. 2024 Jul 3;11(1):65. doi: 10.1186/s40643-024-00779-z (PMC11222365; doi:10.1186/s40643-024-00779-z)

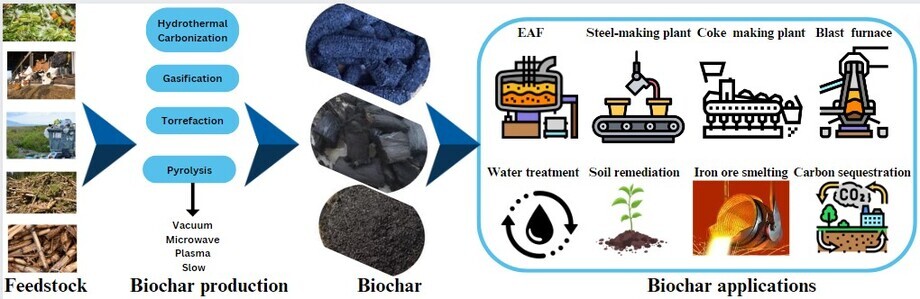

Supplement: Supplementary file 1 — Supplementary Material 1 [file 40643_2024_779_MOESM1_ESM.jpg]
